# Microplastics in freshwaters and drinking water: Critical review and assessment of data quality

**DOI:** 10.1016/j.watres.2019.02.054

**Published:** 2019-05-15

**Authors:** Albert A. Koelmans, Nur Hazimah Mohamed Nor, Enya Hermsen, Merel Kooi, Svenja M. Mintenig, Jennifer De France

**Affiliations:** a Aquatic Ecology and Water Quality Management Group, Wageningen University, the Netherlands; b Copernicus Institute of Sustainable Development, Utrecht University, the Netherlands; c KWR Watercycle Research Institute, Nieuwegein, the Netherlands; d World Health Organisation (WHO), Avenue Appia 20, 1211, Geneva, Switzerland

**Keywords:** Microplastics, Drinking water, Waste water, Surface water, Human health

## Abstract

Microplastics have recently been detected in drinking water as well as in drinking water sources. This presence has triggered discussions on possible implications for human health. However, there have been questions regarding the quality of these occurrence studies since there are no standard sampling, extraction and identification methods for microplastics. Accordingly, we assessed the quality of fifty studies researching microplastics in drinking water and in its major freshwater sources. This includes an assessment of microplastic occurrence data from river and lake water, groundwater, tap water and bottled drinking water. Studies of occurrence in wastewater were also reviewed. We review and propose best practices to sample, extract and detect microplastics and provide a quantitative quality assessment of studies reporting microplastic concentrations. Further, we summarize the findings related to microplastic concentrations, polymer types and particle shapes. Microplastics are frequently present in freshwaters and drinking water, and number concentrations spanned ten orders of magnitude (1 × 10^−2^ to 10^8^ #/m^3^) across individual samples and water types. However, only four out of 50 studies received positive scores for all proposed quality criteria, implying there is a significant need to improve quality assurance of microplastic sampling and analysis in water samples. The order in globally detected polymers in these studies is PE ≈ PP > PS > PVC > PET, which probably reflects the global plastic demand and a higher tendency for PVC and PET to settle as a result of their higher densities. Fragments, fibres, film, foam and pellets were the most frequently reported shapes. We conclude that more high quality data is needed on the occurrence of microplastics in drinking water, to better understand potential exposure and to inform human health risk assessments.

## Highlights

• Fifty studies on microplastics in drinking water and freshwater were reviewed.
• This included lake water, groundwater, tap water and bottled drinking water.
• The quality of the studies was quantitatively assessed.
• Four out of fifty studies scored positive on all quality criteria.
• To understand human health implications, more high quality data is needed.

## Graphical abstract

**Figure:**
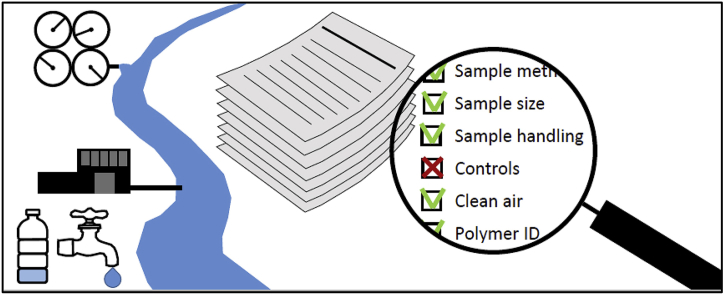

## 1 Introduction

Microplastics are generally characterised as water-insoluble, solid polymer particles that are ≤5 mm in size (Bergmann et al., 2015). A formal definition for the lower size boundary does not exist, but particles below 1 μm are usually referred to as nanoplastics rather than microplastic (Koelmans et al., 2015). Although microplastics are often detected in the environment, the risks they pose are debated and largely unknown. One key challenge in assessing the risks of microplastics to humans and the environment relates to the variability of the physical and chemical properties, composition and concentration of the particles. Further, microplastics in the environment are difficult to identify and standardized methods do not exist (Mintenig et al., 2018). The dominant source of microplastics often is the fragmentation of larger plastics or product wear, however the rate of fragmentation under natural conditions is unknown (Eerkes-Medrano and Thompson, 2018). These challenges and unknowns hamper the prospective assessment of exposure and risk (Koelmans et al., 2017). In this uncertain field, regulatory efforts to examine microplastic safety have been raised (SAM, 2018a, b).

The presence of microplastics has been reported for air samples, food and drinking water (EFSA, 2016; Gasperi et al., 2018; Lusher et al., 2017; Van Cauwenberghe and Janssen, 2014; Wright and Kelly, 2017; Yang et al., 2015) and recently, the implications of microplastics for human health have been reviewed (Wright and Kelly, 2017). Although microplastic exposure via ingestion or inhalation could occur, the human health effects are still unknown. If inhaled or ingested, limited data from animal studies suggest that microplastics may accumulate and cause particle toxicity by inducing an immune response (Deng et al., 2017; Gasperi et al., 2018). Chemical toxicity could occur due to leaching of plastic-associated chemicals (additives as well as adsorbed toxins) (Diepens and Koelmans, 2018; SAPEA, 2019). Such effects are likely to be dose-dependent, however knowledge of exposure levels is currently lacking. Furthermore, biofilms growing on microplastics may be a source of microbial pathogens (GESAMP, 2016). Hence, although there are potential chemical, particle and microbial hazards associated with microplastics, current exposure levels, including through drinking water need to be assessed first.

The ubiquity of microplastics of all sizes in surface water, groundwater and wastewater (SAPEA, 2019), has raised the question if pollution of drinking water occurs. To date, there is only a limited number of studies that address this issue and they indeed reported the presence of microplastics in tap water and bottled water (Kosuth et al., 2018; Mason et al., 2018; Mintenig et al., 2019a, Mintenig et al., 2019b; Schymanski et al., 2018). Some of these studies triggered a great deal of attention in the scientific community as well as the media, putting the issue of human exposure to microplastics via drinking water high on the agenda of public health agencies worldwide. More broadly, ensuring safe drinking water is high on the political agenda, with a dedicated target on safe and affordable drinking water under the Sustainable Development Goals (SDG 6) (WHO and UNICEF, 2017).

To date, about 50 studies exist that provide concentration data for microplastics in drinking water or its freshwater sources, i.e., surface water and groundwater, as well as (indirectly) wastewater. These studies provide data for specific types of water, but methods of sampling, isolating, purifying and identifying microplastics vary enormously among studies. A systematic review of methodologies used and study characteristics is currently lacking. There are several scoping reviews that emphasise the relevance of microplastics in freshwaters (Eerkes-Medrano and Thompson, 2018; Li et al., 2018; Wagner et al., 2014) or that specifically discuss processes or models in freshwaters (Kooi et al., 2018). We are aware of only a limited number of reviews that touch upon methodologies and concentration data (Eerkes-Medrano and Thompson, 2018; Li et al., 2018).

Besides variation in methodologies used and concentrations reported, existing studies are likely to vary with respect to the level of quality assurance deployed. The quality of microplastic research has been debated recently (Burton, 2017; Connors et al., 2017; Koelmans et al., 2016) and has been quantitatively assessed for studies on microplastic ingestion by biota (Hermsen et al., 2018). However, a critical review of studies reporting concentration data in freshwater and drinking water, which also evaluates the quality of applied sampling methods, microplastic extraction and identification steps, is currently lacking.

For chemical risk assessments in a regulatory context, quality criteria have been set in order to be able to evaluate the reliability of data from toxicological studies (Kase et al., 2016; Klimisch et al., 1997; Schneider et al., 2009). Such criteria contribute to the harmonization of the hazard and risk assessments of chemicals across different regulatory frameworks. Recently, Hermsen et al. proposed a weight-of-evidence scoring method for studies of microplastic ingestion by marine biota (Hermsen et al., 2018). This method defined minimum quality criteria for various aspects of the analytical procedure, such as sampling, sample treatment, use of controls and polymer identification. It assigns a score for each aspect and provides a total reliability score for data reported in a study. Such a method can also be developed for the analysis of microplastics in freshwater samples, and can be applied to quantify the relative reliability of reported concentration data.

The aim of the present paper is to critically review the available literature on microplastics in drinking water and its freshwater sources, from a quality assurance perspective and by using a quantitative approach. Wastewater studies were also assessed as these are discharged into the environment. Further aims are to review data on concentration, polymer type, shape and size distribution data across studies. Guidance is provided to improve the quality of future occurrence studies.

Our paper is organised as follows. We first present the key areas that should be assessed to determine the reliability of studies. These areas are presented in separate sections and are: sampling method, sample size, sample processing and storage, laboratory preparation and clean air conditions, negative controls, positive controls, sample treatment and polymer identification. For each of these areas we discuss quality assurance aspects, considerations for scoring, and present the assessment scores for each of these criteria. Subsequently, the combined overall reliability scores are discussed, followed by a discussion on implications for human health risk assessments. In the section thereafter we discuss the outcomes of the reviewed studies. An overview of the concentrations, shapes and polymer types measured is provided and trends are discussed with respect to sample type, location or system characteristics. Finally, we provide recommendations to improve the analysis of microplastics in water samples and summarize the key conclusions.

## 2 Methods

### 2.1 Literature search

Fifty-five records from fifty studies reporting microplastic concentrations in drinking water (2 tap, 3 bottled water) or its freshwater sources (1 groundwater, 30 surface water, 18 wastewater) were reviewed. Some studies reported data on microplastics in more than one water type. Most papers were retrieved from the Scopus database. Search strings used were *microplastic AND (bottle OR surface OR tap OR wastewater OR groundwater).* Three studies were from the grey i.e. not peer-reviewed literature and were found via Google searches, using the same or similar key word combinations. Searches were performed until August 2018. Only those studies that reported original concentration data were reviewed.

### 2.2 Quantitative quality assessment

The reliability of data in studies was evaluated based on criteria originally developed for microplastic in biota samples by Hermsen et al. (2018), and surface water samples by Mintenig et al. (2019a, in prep.). The present approach further refines the method to different categories of water samples, including tap or bottled drinking water, surface water, groundwater and wastewater. The method uses nine crucial criteria, which are detailed below. Criteria relate to those that are common in analytical chemistry, such as reproducibility of described methods, precision, accuracy and sensitivity, which together determine the robustness of an applied method. Reproducibility does not imply that another researcher would obtain the same result, which is due to the variability in conditions inherent to nature. Reproducibility in the context of analytical chemistry refers to minimizing the contribution of random or systematic error to the total observed variability. For each criterion a value of 2 (reliable), 1 (reliable to a limited extent) or 0 (unreliable) is assigned. A ‘Total Accumulated Score’ (TAS) is calculated by adding scores for individual criteria (maximum 18 points) (Tables 1, S2, S3). For data to be considered sufficiently reliable, a study should preferably have no ‘zero’ values for any of the individual scores (Hermsen et al., 2018).

### 2.3 Study characteristics

For each study the following characteristics were summarized in tabular form (Table S1): Reference, Country (area), Source (water type), Treatment applied (for wastewater treatment plants (WWTP) or drinking water treatment plants (DWTP), bottled and tap water), Sampling date, Size/shape (of microplastics detected), Polymer types (of microplastics detected), Chemicals (analysed on microplastic), Value (of microplastics detected in water sample), Quality assurance applied (detection limit, positive controls, negative controls), Sampling method, Analysis method, Comments. Raw concentration data were pooled per water type: WWTP influent, WWTP effluent, lake, river, canal, groundwater, untreated and treated tap water, and bottled water, and analysed for means, ranges and significance of differences among the water types. As data were not normally distributed, the differences were assessed with the Mann-Whitney-Wilcoxon test with Bonferroni correction.

## 3 Results and discussion

### 3.1 Quality assessment of studies reporting data on microplastics in water samples

In this section, methodological aspects are reviewed in subsections and the final total quality scores are presented and discussed. Following Hermsen et al. (2018), for each aspect, scoring criteria are provided and each criterion is explained and justified (Table S2). Such a score based, quantitative evaluation does not result in an absolute judgment but is an indicator of the reliability of these studies for monitoring purposes and to inform risk assessments of microplastics in the drinking water supply chain. The quality criteria provided here are considered adequate for the present assessment, yet may develop over time with increased experience in sampling and analysing microplastics and better understanding of global concentrations. Here we review the general trends; for details on specific studies the reader is referred to Tables S1 and S3.

#### 3.1.1 Sampling methods

Sampling methods were reviewed to understand the variety of approaches utilized, to assess whether sampling was described in sufficient detail, and to be able to define quality assessment criteria for sampling (Tables S1 and S2). Surface water is sampled by pumping, trawling or filling bottles or buckets, followed by sieving to isolate particles of the desired size range (Table S1) (Li et al., 2018). For wastewater, samples are either grabbed with bottles, pumped directly or collected with automatic composite samplers, then sieved, whereas tap and bottled water are directly sieved. Residues in nets or sieves are typically flushed into glass or metal jars or bottles. To obtain a maximum score of 2, the date, location and materials used should be reported. Specific further criteria were defined for wastewater, surface water, untreated and treated tap water and bottled water. For wastewater, the applied treatment type should be mentioned as this can impact the microplastic concentrations and should be considered when assessing retention or removal efficiencies of individual technologies. For the same reason, this should be done when taking samples on DWTPs. For surface water, the depth of sampling should be reported, as this may affect concentration (Kooi et al., 2018). For tap water, when the aim is to assess concentration in general, running the tap before sampling is recommended (e.g. 1 min) in order to avoid incidental contamination from air (Wesch et al., 2017), unless it is specifically mentioned that the aim is to measure the first portion of the water, e.g., the first glass. Furthermore, flowrate and source of tap water (e.g., storage tank, groundwater, surface water) should be reported, as this may be relevant for data interpretation. For the same reason, for bottled drinking water, the source, batch production lot and bottled water type (sparkling vs still water) should be specified. To maximize particle recovery from the bottle, the sample should be shaken before filtration and the emptied bottle should be flushed three times with filtered water. A score of 1 was assigned if a study provided a subset of the required characteristics (e.g. date, location), but is still fairly reproducible. About half of the studies score 2 on this criterion whereas only three studies score 0.

#### 3.1.2 Sample size

Different factors were considered when recommending an optimum water volume to be sampled. For microplastics, the limit of detection can be seen as the methods’ capability of reliably detecting at least one particle with statistical rigor. A sample volume that is too low reduces the chance of finding particles, reduces the power of a study and increases the margin of error. This means that detection limits benefit from large sample volumes. Similar approaches i.e. sufficient sample size are used when analysing chemicals in environmental matrices (Einax et al., 2004). However, for samples with particles, samples should be small enough to prevent clogging of filters or sieves. This means that recommendations for sample sizes will differ for different water types. Because the actual concentration cannot be predicted, occurrence of non-detects or filter clogging can never be fully prevented.

**Fig. 1:**
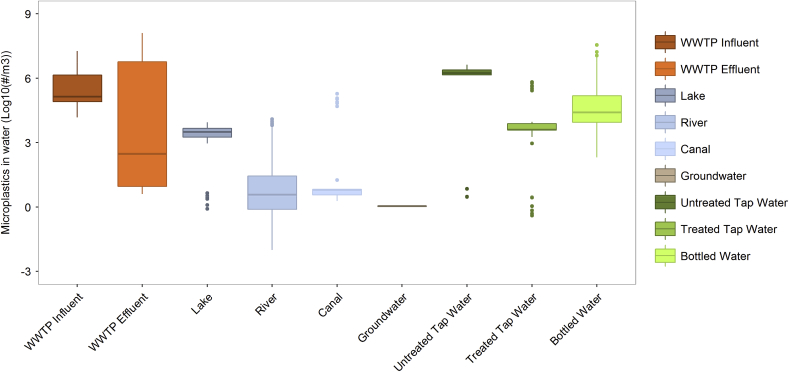
Box and whisker plot showing median and variation in microplastic number concentrations in individual samples taken from different water types. Data relate to individual samples unless only means were reported, in which case the mean value was taken into account n times, with n being the number of samples which the mean was based on. References included: (Estahbanati and Fahrenfeld, 2016; Faure et al., 2015; Fischer et al., 2016; Hoellein et al., 2017; Kosuth et al., 2018; Leslie et al., 2017; Magnusson and Norén, 2014; Mason et al. 2016a, 2018; McCormick et al. 2014, 2016; Michielssen et al., 2016; Mintenig et al., 2019b; Oβmann et al., 2018; Pivokonsky et al., 2018; Rodrigues et al., 2018; Schymanski et al., 2018; Simon et al., 2018; Talvitie et al. 2015, 2017a, 2017b; Vollertsen and Hansen, 2017; Wang et al. 2017, 2018; Ziajahromi et al., 2017), with n = 27. For statistical significances of differences among water types, see Table S4.

Detection limits also depend on the particle size range aimed for in a study. Various studies have shown that smaller particles are more abundant (Cabernard et al., 2018), implying that smaller sample volumes are required when exclusively examining small microplastics that are analytically challenging to detect (e.g., <100–300 μm). However, if such a study would also aim to detect larger microplastics accurately, a large volume would still be required. Establishing sample volume recommendations for studies primarily aiming for larger (roughly > 300 μm) microplastics, should consider both expected microplastic concentrations for a given water type and practical considerations. Most studies reviewed belong to this category that aimed to detect also larger microplastics. In surface water, > 300 μm microplastic concentrations span a wide range of concentrations; roughly 1 × 10^−3^ to 10 particles per litre (Fig. 1). Because of the low concentrations and ease of obtaining large volumes from surface waters, we set 500 L as a minimum sample volume for surface water. However, given the often very low particle number concentrations in some lakes and rivers, a volume greater than 500 L is recommended for remote locations.

For tap water (range 1 × 10^−4^ to 100 particles per litre), a greater sample volume is proposed compared to surface water. We advise a minimum volume of 1000 L, because of the concentrations that can be very low (Mintenig et al., 2019b), uncertainties with the representativeness of this range given the low number of studies identified, and ease of sample collection. For bottled water, there were also a limited number of studies available. Yet they all demonstrate presence of at least several particles per litre, such that even a minimum of 1 L would be defensible in case a 1 L bottle would be the study unit and only very small particles (<100 μm) would be targeted. However, the study unit in such studies is often the brand or production lot, and also larger particles are targeted, in which case we recommend to sample >10 L for a more representative result. As bottled water usually is provided in volumes smaller than 10 L, this would imply the need to either analyse multiple bottles or to treat the total volume of multiple bottles as one sample. For WWTP influents where concentrations of particles are expected to be higher (Fig. 1), a sample volume of 1 L is considered sufficient. For WWTP effluent, a sample volume greater than 500 L is recommended, or a reported clogging of the sieve e.g. (Carr et al., 2016; Mintenig et al., 2017; Vollertsen and Hansen, 2017; Ziajahromi et al., 2017). These volumes mentioned would lead to roughly 5 to 500 particles detected, which is considered sufficiently representative if the detection limit would be 1 particle as mentioned above. Use of these volumes would receive a maximum score of 2. However in some cases lower volumes have been used with good reason and may still yield fair results. In these cases a score of 1 is assigned (Table S2). Studies that explicitly aim for only smaller particles can use smaller volumes as long as detection limits are met, and still receive the maximum score.

#### 3.1.3 Sample processing and storage

For the transfer of a primary sample (e.g. material in a net or sieve) to a storage bottle, or for preservation or storage of samples before reaching the laboratory, certain criteria need to be met. Some studies rinse jars, bottles or other materials with targeted water e.g. (Kosuth et al., 2018; Talvitie et al., 2015). However, particles from that rinsing water could easily stick to surfaces and remain, which thus would lead to contamination of the actual sample. Ideally, sample containers should be rinsed in the laboratory with filtered water before bringing them to the field. In general, samples should be stored shortly after sampling and further handling avoided before arriving in the laboratory. When sampling, use of plastic materials should be avoided as much as possible to again minimize contamination. Many studies use a fixative like ethanol, formalin or methyl aldehyde (Anderson et al., 2017; Baldwin et al., 2016; Eriksen et al., 2013; Fischer et al., 2016; Mason et al., 2016a; Su et al., 2016; Wang et al., 2018; Xiong et al., 2018; Zhang et al. 2015, 2017). However, the effects of the fixative on different types of plastic should be evaluated before application, or studies should report evidence from the literature (Hermsen et al., 2018). Ethanol and formalin for instance, have been shown not to affect polymer characteristics (Courtene-Jones et al., 2017). Some of the studies reviewed here used volunteers for sampling and sample processing (Christiansen, 2018; Kosuth et al., 2018). Citizen science (CS) approaches have been used in environmental monitoring and are increasingly being used in research on plastic debris (Liboiron et al., 2016; Syberg et al., 2018). It has been argued that this may improve risk perception within society and therefore improve the foundation for timely and efficient societal measures (Syberg et al., 2018). There is also an economic incentive to collect data with volunteers rather than by paid professionals, and some monitoring research would even be impracticable if data were not collected by volunteers (Brett, 2017). However, concerns with respect to the quality of CS have been raised, and validation studies have shown that the reliability of CS based data is highly uncertain (Brett, 2017). Other than for macroplastics, quality assurance for sampling and sample processing of microplastics is technically demanding and the error rate can be expected to be higher for volunteers than for professionals. Since no CS validation studies for microplastics sampling and analysis exist to date, it is not clear to what extent the quality of data is affected by having some of the crucial steps performed by non-professionals. Therefore, as scientific quality assurance is the primary perspective of this paper, use of volunteers for major parts of the sampling work was considered less reliable, leading to a score of 1 in case of validation of the adequacy of the protocols, and 0 in all other cases for this criterion.

#### 3.1.4 Laboratory preparation

Contamination of samples due to airborne polymer particles and fibres has been described as a major problem in microplastic analysis (Hermsen et al. 2017, 2018; Torre et al., 2016; Vandermeersch et al., 2015; Wesch et al., 2016). Therefore, to avoid contamination and prior to actual sample preparation and analysis, certain measures need to be taken. These include avoiding synthetic components in clothing, wearing of cotton lab coats, and pre-rinsing and cleaning of all materials used as well as laboratory (bench, laminar flow cabinet) surfaces. If precautions were not fully reported but sufficient blanks (i.e., three blanks, see section ‘negative controls’ below) were included to keep track of background contamination, then a score of 1 was assigned (Table S2).

#### 3.1.5 Clean air conditions

To avoid contamination with airborne microplastic particles or fibres, sample handling should be performed in a laminar flow cabinet or in a clean air laboratory to receive the maximum score (Hermsen et al., 2018). Recent studies are increasingly using such conditions (Mason et al., 2018; Oβmann et al., 2018; Schymanski et al., 2018; Wang et al., 2018; Zhang et al., 2017). In case clean air conditions were not used but covering of samples and sufficient blanks were reported, a score of 1 was assigned (Cable et al., 2017; Dris et al. 2015, 2018b; Miller et al., 2017; Mintenig et al., 2019b; Pivokonsky et al., 2018).

#### 3.1.6 Negative controls

To verify and correct for contamination or to demonstrate absence of contamination, replicated (n ≥ 3) procedural blanks need to be analysed. All reviewed studies reported particles counts; if the variability of contamination was quantified, and if it was clearly indicated that actual sample results were corrected for blank values, a score of 2 was assigned. Some precautions are less reliable but still provide some useful information on the level of contamination, like the filtration of air, or the sole examination of petri dishes/soaked papers placed next to the samples (Cable et al., 2017; Dris et al. 2015, 2018b; Estahbanati and Fahrenfeld, 2016; Hendrickson et al., 2018; Lares et al., 2018; Mani et al., 2015; McCormick et al., 2016; Rodrigues et al., 2018; Simon et al., 2018; Ziajahromi et al., 2017). If these precautions were taken, a score of 1 was assigned.

#### 3.1.7 Positive controls

Losses of particles may occur during various steps of sampling, sample preparation and analysis and it is recommended to quantify losses using positive controls. Estahbanati and Fahrenfeld (2016) assessed particle losses during sampling with nets, by adding plastic particles in distilled water. Subsequent sample handling in the laboratory often includes complex steps to remove organic matter from samples (see ‘sample treatment’ below), particularly from WWTP influent or effluent or surface waters. To verify a sufficiently high recovery of particles during filtration, digestion, transfer and analytical identification steps, representative replicated positive controls (n ≥ 3) should be performed (Hermsen et al., 2018). If recoveries are low yet reproducible, the reported counts should be corrected for this incomplete recovery. Positive controls should be conducted for the targeted microplastics, covering different size classes and polymer types. Microplastic sizes span a wide range and it cannot be assumed that recoveries are constant across the range of sizes and polymer types. In practice, it is important to at least use small enough microplastics as controls, as these are more difficult to recover. In some cases, larger microplastics still require separate controls, especially when different methods are applied. For instance, the method used by Mason et al. (2018) for particles smaller than 100 μm was different from that for particles larger than 100 μm, whereas positive controls were only performed for the smaller particles. Only three studies provided full data on positive controls (Simon et al., 2018; Vollertsen and Hansen, 2017; Wang et al., 2018) and received maximum scores, indicating that it is not yet a very common practice. Other studies conducted positive controls but with no or insufficient replicates (Di and Wang, 2018; Dyachenko et al., 2017; Hendrickson et al., 2018), or only for one step in the analysis (Rodrigues et al., 2018), or for part of the targeted size range (Mason et al., 2018) and received a score of 1.

#### 3.1.8 Sample treatment

To assure the quality of visual inspection and subsequent polymer identification, which is especially critical for <300 μm particles and to enable the usage of more advanced identification techniques (see section ‘polymer identification’), a sample digestion step should be performed for surface and WWTP water samples in order to score 2 points. Tap and bottled water do not require a digestion step and thus were always assigned 2 points on this criterion. Digestion should be done under conditions that do not affect the microplastics weights, counts or shapes. In the context of biota analysis, use of potassium hydroxide (KOH) or enzymes has been demonstrated to be acceptable (Catarino et al., 2016; Cole et al., 2014; Kühn et al., 2017; Munno et al., 2018). The reviewed studies here commonly used hydrogen peroxide (H_2_O_2_) which is known to affect some polymers (Hurley et al., 2018). However its effects have been demonstrated to be minimal within an exposure of 48 h (Löder et al., 2017) and was therefore deemed acceptable. Several studies kept the temperature around 35–45 °C, e.g. by using a cooling or ice bath (Simon et al., 2018), however sometimes higher temperatures up to 75 °C (Anderson et al., 2017; Baldwin et al., 2016; Estahbanati and Fahrenfeld, 2016; Hendrickson et al., 2018; Hoellein et al., 2017; Pivokonsky et al., 2018) or even 80 °C were used in some of the digestion steps (Vermaire et al., 2017), or even 90 °C for drying (Estahbanati and Fahrenfeld, 2016; Hendrickson et al., 2018; Ziajahromi et al., 2017). Effects of temperature in combination with various digestion chemicals were studied by Munno et al. (2018). Based on comparison of data on polymer mass losses during heating and digestion, the authors concluded it was best to stay below 60 °C. We set 50 °C as the safe upper limit, and as a criterion to assign a maximum score as a precautionary measure and since many of the reviewed studies were below 50 °C. Digestion without such considerations of mass losses was assigned a score of 1. A score of 1 was also assigned for surface water when it was reported to be very clear and clean even without digestion applied. Furthermore, studies that did not apply digestion but explicitly were aiming for the detection of ≥300 μm particles only, were assigned a score of 1 (Hermsen et al., 2018).

#### 3.1.9 Polymer identification

To assure reliable assessment of plastic particles, the polymer identity needs to be confirmed, preferably by using (micro) FTIR or Raman spectroscopy, pyrolysis-GCMS or TGA-GCMS techniques (Hermsen et al., 2018; Löder and Gerdts, 2015; Mintenig et al., 2018). Although subsampling should be avoided, these techniques are so laborious that representative sub-sampling is often required. Best practice for subsampling and subsequent polymer identification will differ for different microplastic size classes and technologies applied (Mintenig et al., 2018). The manual sorting and subsequent identification of microplastics has a bias compared to the identification of particles enriched on filters with FTIR or Raman microscopy (i.e., avoid missing transparent or small particles), and is therefore discouraged when analysing particles <300 μm. For manually sorted particles, following Hermsen et al. (2018), we argue that analysis of all particles is feasible and therefore recommended if the numbers of pre-sorted particles *per study* are <100. For particle numbers >100, 50% should be identified, with a minimum of 100 particles. If polymer identities are reported on a *per sample* basis, we also advise to analyse all particles found, however with a minimum of 50. This minimum is considered reasonable to represent the variety of particle shapes and polymer types in environmental samples. Anyway, for such hand-picked representative subsets, studies generally still should describe how representativeness was assured. For smaller micropastics and when applying FTIR or Raman microscopy, the representativeness of subsampling (the area of a filter that was measured) is relatively easy to assess. Particularly when coupling a focal plane array detector to the microscope, many more particles (especially the small and transparent particles) can be assessed in one analysis. Although measurement times can be long, at least 25% of the filter needs to be analysed (Mintenig et al., 2017; Redondo-Hasselerharm et al., 2018). If these criteria for number of particles and/or percentage of the filter are met, a score of 2 is assigned. If polymers were identified for a too low number of particles or on a smaller part of the filter, a score of 1 was assigned. Also, if SEM-EDS or - EDX was applied to distinguish polymers from non-polymeric materials (Anderson et al., 2017; Cable et al., 2017; Mason et al., 2016b; Su et al., 2016), a score of 1 was assigned

#### 3.1.10 Overall reliability of method aspects and studies

For each study, we assessed against all quality criteria and calculated a total accumulated score (TAS) (Table S3). Whereas the maximum achievable TAS score is 18, average (min – max) TAS scores were 13.7 (13–14) for bottled water, 11.5 (8–15) for treated tap water, 12.5 (11–14) for DWTP water, 7.9 (4–15) for surface water, and 7.3 (3–13) for waste water studies, respectively (Table 1). This ranking in average scores for the different water types probably reflects the relative ease of analysing these different water types. For instance, bottled and tap water require no digestion, which means that 2 points were always assigned to the sample digestion criteria. It should be noted though that the number of studies examining DWTP and treated tap water (each n = 2), and bottled water studies (n = 3) was very low, rendering the averages to be less rigorous. On average, studies were assigned roughly half (8.41/18) of the maximum score for data quality, a result which is very similar to the average score assigned to studies reporting data on ingestion of microplastic by biota (Hermsen et al., 2018).

**Table 1:** Overview of individual and accumulated scores^a^ of papers reporting microplastic concentrations in surface water and drinking water.

Author	Type	Sampling methods	Sample size	Sample processing and storage	Lab preparation	Clean air conditions	Negative controls	Positive controls	Sample treatment	Polymer ID	Total Accumulated Score^b^ (TAS, max = 18)
Mason et al. (2018)	**Bottle**	1	2	2	1	2	2	1	2	1	**14**
Schymanski et al. (2018)	**Bottle**	1	1	2	2	2	2	0	2	2	**14**
Oβmann et al. (2018)	**Bottle**	1	1	2	2	2	2	0	2	1	**13**
Mintenig et al., 2019a, Mintenig et al., 2019b	**Tap**	2	2	2	2	1	2	0	2	2	**15**
Kosuth et al. (2018)	**Tap**	0	0	0	2	2	2	0	2	0	**8**
Mintenig et al., 2019a, Mintenig et al., 2019b	**DWTP**	2	1	2	2	1	2	0	2	2	**14**
Pivokonsky et al. (2018)	**DWTP**	1	1	2	1	1	2	0	1	2	**11**
Mintenig et al., 2019a, Mintenig et al., 2019b	**Ground**	2	1	2	2	1	2	0	2	2	**14**
Wang et al. (2018)	**Surface**	2	1	1	2	2	2	2	2	1	**15**
Hendrickson et al. (2018)	**Surface**	2	1	2	1	1	1	1	1	1	**11**
Di and Wang (2018)	**Surface**	2	0	2	2	0	0	1	2	1	**10**
Mani et al. (2015)	**Surface**	2	2	1	1	1	1	0	1	1	**10**
Wang et al. (2017)	**Surface**	1	0	1	2	1	2	0	2	1	**10**
Baldwin et al. (2016)	**Surface**	2	1	1	1	1	2	0	1	0	**9**
Cable et al. (2017)	**Surface**	2	1	1	1	1	1	0	1	1	**9**
Dris et al. (2018a)	**Surface**	2	2	0	1	1	1	0	1	1	**9**
Lares et al. (2018)	**Surface**	1	0	1	2	1	2	0	1	1	**9**
Rodrigues et al. (2018)	**Surface**	2	2	1	1	0	1	0	1	1	**9**
Su et al. (2016)	**Surface**	2	1	1	1	1	1	0	1	1	**9**
Zhang et al. (2017)	**Surface**	2	1	1	1	2	0	0	0	2	**9**
Dris et al. (2015)	**Surface**	2	1	2	1	1	1	0	0	0	**8**
Estahbanati and Fahrenfeld (2016)	**Surface**	2	2	1	0	0	1	1	1	0	**8**
Hoellein et al. (2017)	**Surface**	2	1	2	0	0	1	0	1	1	**8**
Mason et al. (2016b)	**Surface**	2	1	1	0	0	2	0	1	1	**8**
Sighicelli et al. (2018)	**Surface**	2	2	1	0	0	0	0	2	1	**8**
Vermaire et al. (2017)	**Surface**	2	1	2	0	0	2	0	1	0	**8**
Xiong et al. (2018)	**Surface**	2	1	0	1	1	1	0	1	1	**8**
Anderson et al. (2017)	**Surface**	2	1	1	0	0	1	0	1	1	**7**
Faure et al. (2015)	**Surface**	1	2	1	1	0	0	0	1	1	**7**
McCormick et al. (2016)	**Surface**	1	1	1	0	0	2	0	1	1	**7**
Miller et al. (2017)	**Surface**	1	0	1	1	1	2	0	0	1	**7**
McCormick et al. (2014)	**Surface**	1	1	1	0	0	2	0	1	0	**6**
Fischer et al. (2016)	**Surface**	2	1	1	0	0	0	0	1	0	**5**
Free et al. (2014)	**Surface**	2	1	1	0	0	0	0	1	0	**5**
Lahens et al. (2018)	**Surface**	1	1	1	0	0	0	0	1	1	**5**
Leslie et al. (2017)	**Surface**	1	0	2	0	1	1	0	0	0	**5**
Eriksen et al. (2013)	**Surface**	2	1	1	0	0	0	0	0	0	**4**
Zhang et al. (2015)	**Surface**	2	1	0	0	0	0	0	0	1	**4**
Mintenig et al. (2017)	**WWTP**	2	2	2	1	1	2	0	1	2	**13**
Ziajahromi et al. (2017)	**WWTP**	2	2	1	1	1	1	1	1	2	**12**
Simon et al. (2018)	**WWTP**	1	1	0	1	1	2	2	2	1	**11**
Lares et al. (2018)	**WWTP**	2	0	1	2	1	2	0	1	1	**10**
Talvitie et al. (2017a)	**WWTP**	2	1	1	1	1	2	0	0	2	**10**
Murphy et al. (2016)	**WWTP**	1	1	2	2	1	1	0	0	1	**9**
Mason et al. (2016a)	**WWTP**	2	2	1	0	0	2	0	1	0	**8**
Vollertsen and Hansen (2017)	**WWTP**	0	2	1	0	0	0	2	1	1	**7**
Carr et al. (2016)	**WWTP**	2	2	1	0	0	0	0	0	1	**6**
Magnusson and Norén (2014)	**WWTP**	2	2	1	0	0	0	0	0	1	**6**
Michielssen et al. (2016)	**WWTP**	2	1	2	0	0	1	0	0	0	**6**
Talvitie et al. (2017b)	**WWTP**	2	0	1	0	0	2	0	0	1	**6**
Vermaire et al. (2017)	**WWTP**	1	0	2	0	0	2	0	1	0	**6**
Dyachenko et al. (2017)	**WWTP**	1	0	1	0	0	0	1	1	1	**5**
Leslie et al. (2017)	**WWTP**	1	0	2	0	1	1	0	0	0	**5**
Dris et al. (2015)	**WWTP**	1	0	0	1	1	1	0	0	0	**4**
Talvitie et al. (2015)	**WWTP**	2	1	0	0	0	1	0	0	0	**4**
Browne et al. (2011)	**WWTP**	0	0	1	0	0	0	0	0	2	**3**

**Average**		**1.57**	**1.02**	**1.20**	**0.77**	**0.64**	**1.18**	**0.21**	**0.93**	**0.89**	**8.41**

a For the scoring criteria, the reader is referred to Table S2.

b TAS values are underlined when all underlying scores are non-zero.

Only four studies received non-zero scores for all criteria. These were the study on surface water by (Wang et al. 2018) (TAS = 15), the study on bottled water by Mason et al. (2018) (TAS = 14), and two studies on wastewater by Ziajahromi et al. (2017) (TAS = 12) and Hendrickson et al. (2018) (TAS = 11). For the ranking of such non-zero studies, a multiplied score X can be calculated (Hermsen et al., 2018), followed by a ^2^Log X transformation in order to obtain a linear scale for a maximum score of 9. This would lead to a score of 6 for the data provided by Wang et al. (2018), a score of 5 for the data provided by Mason et al. (2018), a score of 3 by Ziajahromi et al. (2017), and a score of 2 for the data provided by Hendrickson et al. (2018). These four studies were published in the years 2017 or 2018, which may reflect recent progress in the quality of applied methods to analyse microplastics in environmental samples. With only four studies having all non-zero scores, it can be concluded that the majority of the reviewed studies (46 studies or 92%) cannot be considered fully complete or reliable on at least one crucial aspect of quality assurance. This does not mean that studies may not be useable or important as a more specific consideration of scores and study outcomes in hindsight, can still make a study very well fit for certain research questions.

Besides insights in methodological differences among individual studies, the scores allow for a cross comparison of reliability differences per criterion (Table 1) (Hermsen et al., 2018). Average scores per criterion were all lower than 2, which means there is room for improvement of quality assurance in this field of research. The average scores per criterion across 55 records were lower than 1 for the criteria *sample treatment* (0.93), *polymer identification* (0.89), *laboratory preparation* (0.77), *clean air conditions* (0.64), and *positive controls* (0.21). Therefore, significant improvements are needed especially for these five out of nine quality aspects. Our analysis further illustrates that besides actual quality assurance, also full reportage of method details is important, to assure traceability and reproducibility of data. Reporting is a quality aspect in itself and some studies may have scored higher had they been reported better. In this respect we recommend to also include detection limits in terms of number and mass concentrations, but also in terms of minimum and maximum detectable particles sizes inherent to the applied methodology.

#### 3.1.11 Implications of quality criteria and reliability of studies for human health risk assessment

Human health risks depend on exposure and it is well known that drinking water is an uptake pathway for microplastics. Consequently, quality in the analysis of microplastics in drinking water and its sources is very relevant to accurately assess risks to human health.

In this respect it should be mentioned that the proposed criteria are related to concentrations in the water, which however may not fully correlate with exposure. For instance, we recommended running the tap before sampling to avoid contamination of the first portion of water, to assure reproducibility of results and further, because many consumers would do this anyway. However, others may not do this and addressing this variability may be relevant for exposure assessment. Exposure to microplastics may also depend on the level of shaking of a bottle before drinking, whereas our criteria recommend shaking in order to maximize the chance that all particles are measured, and to assure reproducibility of the analysis. Exposure in drinking water can additionally be influenced by direct contamination of drinking water through contact with air, but to better understand contamination that is coming directly from the water supply and to support comparability and reproduciblity, we recommend procedures to prevent airborne contamination. Finally, exposure to microplastics would also include uptake via inhalation or food (Wright and Kelly, 2017), which is not covered in this paper that only addresses drinking water and its sources.

The fact that high quality data are limited also has implications for human health risk assessment, which considers both exposure as well as health effects. Only four out of 50 studies (which were published in 2017 and 2018) were of such a level of reliability (i.e. having no zero scores) that they could be used confidently for an exposure assessment. Importantly, of these four studies, the recent study on microplastic particles in bottled drinking water (Mason et al., 2018) would be highly relevant for human health risk assessment, based on the criteria used here, although the study only had maximum scores in 5 out of 9 criteria. Therefore, this uncertainty in the overall exposure data precludes the ability to conduct a robust risk assessment, whether related to particle toxicity, chemical toxicity or microbial toxicity. We therefore conclude that more high quality data is needed on the occurrence of microplastics in drinking water to more confidently assess potential exposure, as a critical piece for understanding the potential human health risks.

### 3.2 Microplastics in freshwater

#### 3.2.1 Global microplastic concentrations in different water types

We reviewed the available literature on microplastics in drinking water, fresh water and wastewater. Monitoring has been conducted in multiple locations in Asia, Australia, Europe and North America. A selection of studies reporting particle number concentrations were used for a further analysis (Figs. 1 and 4), if they reported means and/or raw data on a volume basis. These microplastic concentrations, reported as number of particles, spanned ten orders of magnitude (1 × 10^−2^ to 10^8^ #/m^3^) across all individual samples and water types, also when excluding wastewaters (Fig. 1). The number of microplastic particles in samples per water type was statistically different (p < 0.05) for all pairwise comparisons of water types, except for the comparisons between ground water and all other water types, WWTP effluent versus (untreated) DWTP and tap water, and WWTP influent versus (untreated) DWTP water (Fig. 1, Table S4). As these concentration data relate to numbers, they do not distinguish between particle size, shape or material type; differences that will be discussed in the sections below. Studies often do not mention a lower nor an upper size limit, or only mention the targeted size class. The data include particles *reported* as microplastics, that is, we did not take out suspect non-polymer particles as identified either by authors themselves or based on our quality assessment discussed above. The range for 50% of the data per water type (the boxes in Fig. 1) is 1–2 orders of magnitude, and quite similar for influent, effluent, lake, river and bottled water data. For canal and tap water only a few studies were available, which may have caused the variation to be much smaller. For bottled water, the number of studies was also low (Mason et al., 2018; Oβmann et al., 2018; Schymanski et al., 2018), however there were many samples (bottled water brands) for this water type available in these studies. The median concentrations per water type vary over four orders of magnitude.

Some general patterns exist in the concentration data (Fig. 1). Surface waters have the lowest concentrations of all water types, with, bottled water closer to the higher end. The lower concentrations observed in surface water, particularly compared to drinking water, is likely attributed to the fact that most surface water studies targeted only larger particles whereas smaller particles are more abundant (Cabernard et al., 2018). WWTP influent shows the highest concentrations based on the median and interquartile range of reported concentrations (Fig. 1) although WWTP studies generally did not monitor small particles. The high concentrations therefore reflect direct domestic inputs and inputs from those diffuse land-based sources that are routed via waste water. WWTP effluent has a lower median compared to WWTP influent, which probably reflects the retention of microplastics in WWTPs. Similarly, untreated tap water has higher concentrations than treated tap water. Concentrations in bottled water are higher than in tap water, which may reflect the higher influx of airborne particles in the factories, which are inherently more locked in, wear from caps or bottle walls after production, or the fact that these studies also included smaller sized particles. For instance, Schymanski et al. (2018) used Raman microscopy and was thus able to identify down to > 5 μm, which also explains the high number concentrations. The general trends observed here (Fig. 1) still remain when only the studies that received highest quality scores are taken into account (Fig. S1). Still, the generalities listed here should be interpreted with caution given the low number of bottled water (n=3), treated tap water (n = 2), (untreated) DWTP water (n = 2) and ground water studies (1), although as noted earlier, there were many bottled water samples available in the limited number of studies.

#### 3.2.2 Microplastic shapes in global freshwaters

**Fig. 2:**
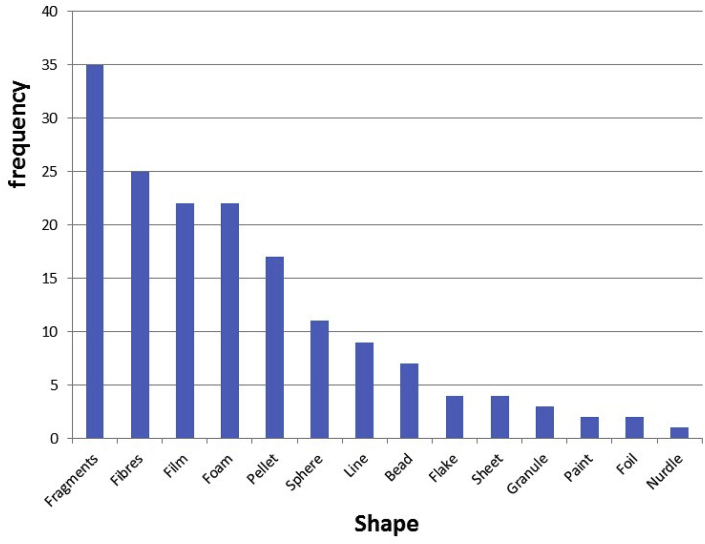
Number of studies reporting a particular shape of microplastic particles (from a total of 55 records).

Microplastics of different shapes were reported. Several factors limit a potential quantitative analysis of reported data on the relative abundance of shapes among water types. First, many studies typically only analysed shapes of a subset of all isolated particles and it is not clear how representative these subsets were when it comes to particle shape. Second, studies targeted different size ranges which also limits their comparability. For instance, fibres are typically small (Cole, 2016), so easily missed when trawling. Third, studies differed in the extent their water samples were representative of the studied water systems or water type, which in turn is affected by spatial and temporal variability. Fourth, although some particles’ shapes were quite well-defined and thus interpreted similarly across studies, some others are more ambiguous, like nurdle, pellet, pre-production pellet, sphere, resin or granule. Nevertheless, we can provide a relatively robust view of the relative importance of particle shapes by showing the frequency of shapes observed across studies (Fig. 2). The reviewed studies (n = 50) reported (in the order of decreasing reporting frequency): fragment, fibre, film, foam, pellet, sphere, line, bead, flake, sheet, granule, paint, foil and nurdle (Fig. 2). We argue that this order also reflects a relative order of importance of shapes, that is, the most frequent shapes detected in a high number of locations globally, as the reviewed studies concerned many different locations on the globe.

#### 3.2.3 Polymer types reported in global studies on freshwater microplastics

**Fig. 3:**
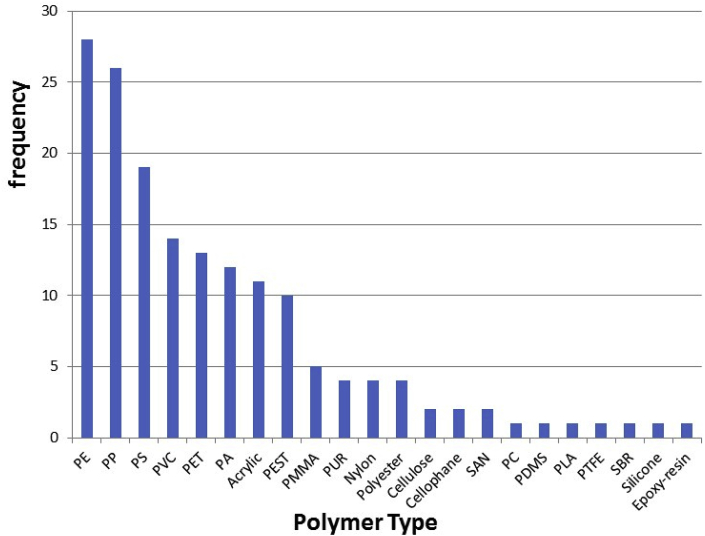
Number of studies reporting a particular polymer type of microplastic particles (32 out of 55 records reported polymer type).

For 32 out of 55 records, polymer types were assessed. Similar to particle shape as discussed above, and rather than discussing relative abundances per study, we consider the relative frequency of reported polymer types observed in water types on a global level. Often, relative abundances per study are not provided, or may not be considered accurate due to limited or biased subsets of particles used for the polymer identification. Most frequently observed polymer types across studies and records are PE ≈ PP > PS > PVC > PET, with Acrylic or acrylic-related compounds, PA, PEST and PMMA reported in five or more records (Fig. 3). The order of the five most abundant polymers can be roughly explained by two factors; global plastic demand and polymer density (Andrady, 2011; Bond et al., 2018). Global plastic demand would cause an order of PE > PP > PVC > PET > PS (Bond et al., 2018; Geyer et al., 2017). However, whereas PE and PP have densities below 1 g/cm^3^ and are buoyant and PS has a density close to that of water, PVC and PET have densities of 1.3–1.7 g/cm^3^. Therefore, a relatively high degree of settling could explain the lower abundances of PVC and PET in the surface water samples mostly assessed here. Specific subsets, i.e. Lakes/Rivers versus WWTP samples were checked for differences in relative abundances of polymer types, but no such differences were found. For a more detailed analysis of polymers reported in studies, the reader is referred to Table S1, which provides all observed polymers on an individual record basis. Recently, Bond et al. (2018) provided a review of polymer abundance data across environmental compartments in Europe, including 3 surface water and 5 WWTP studies. Instead of providing the reporting incidence across a large number of global studies, they averaged relative abundances reported across these 8 European studies, yet found the same order of abundances for the 5 most dominant polymers.

**Fig. 4:**
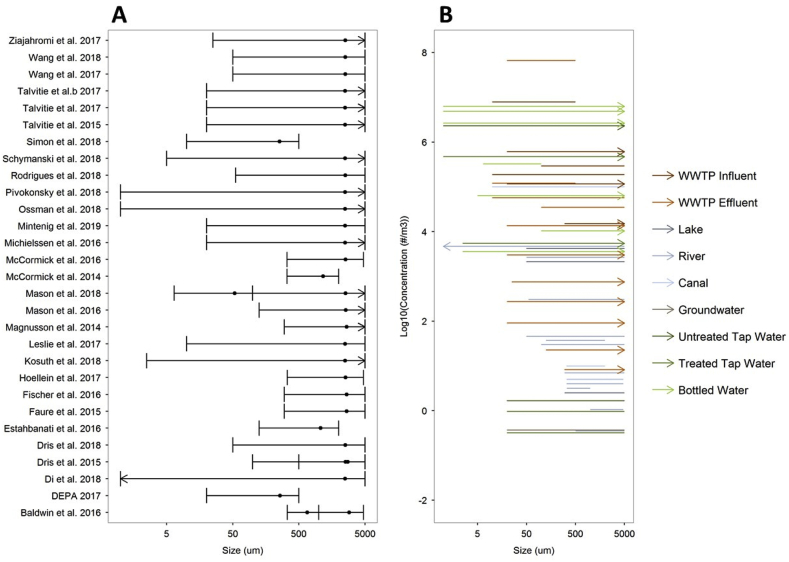
Size ranges used (A) and number concentrations per size range reported (B) in studies on microplastics in drinking, surface and waste waters (referenced in Fig. 1). Arrows indicate that no upper or lower size limit was specified, in which case values of 5 mm or 1 μm were assigned, respectively. Panel A: Size ranges per study are ordered alphabetically per author name. Data points represent the average of the size range. Panel B: reported concentrations as a function of size range. Colours of arrows (Panel B) correspond to colours of the box and whiskers in Fig. 1. (For interpretation of the references to colour in this figure legend, the reader is referred to the Web version of this article.)

#### 3.2.4 Sizes of microplastic particles

Studies generally did not report sizes or size distributions relating to individual particles, which precludes a meta-analysis of particle size across studies. However size classes were reported (Table S1) as well as the number of particles observed per size class. Still, this does not allow for a meaningful quantitative analysis, because the size bins vary widely across studies (Fig. 4A). Furthermore, often lower or upper size limits are not specified so that it is not clear to what size class reported number concentrations actually relate. Instead of plotting the reported size ranges across studies (Fig. 4A), reported ranges can be plotted against mean particle number concentrations (Fig. 4B). The latter graph clearly shows that studies aiming for smaller particles, like some of the bottled water and tap water studies, generally find the higher particle number concentrations.

## 4 Conclusions

We conclude that based on the limited number of high quality studies identified, standardization of microplastic analysis in water is needed. Quality assurance criteria that require the most improvements are sample treatment, polymer identification, laboratory preparation, clean air conditions and positive controls. In addition to ensuring that individual studies are of higher quality in order to achieve more confidence in study findings, standardized methods will allow reproducibility and comparability of results and will lead to the quality of data that are needed to conduct risk assessments. Among water types, reported microplastic concentrations differed widely, but the fact that studies target different size classes contributes to this variability. Despite the quality limitations, our analysis confirmed that microplastic is frequently present in freshwaters and drinking water. There is a high need to improve the analysis of very small microplastics, and to identify them in different water samples. Fragments, fibers, film, foam and pellets were the most frequently found microplastic shapes in surface water samples. Relative abundance of polymer types found across studies reflected plastic production and polymer densities. Conclusions on size comparisons among studies and water types are difficult to draw due to the aforementioned differences in targeted particle sizes. More studies are needed to better understand occurrence, shape, polymer types, and particle sizes, particularly for the small plastic particles.

## Declarations of interest

None.

## Conflicts of interest

There is no conflict of interest.

## Author agreement

AAK and JDF designed the study. NHMN, EH, MK, SM and AAK performed the study. AAK wrote the article. NHMN, EH, MK, SM and JDF commented on draft versions of the article. All authors have approved the final article.

## Disclaimer

The authors alone are responsible for the views expressed in this publication and they do not necessarily represent the views, decisions or policies of the World Health Organization.
