# Supplementary material for: Microplastics in freshwaters and drinking water: Critical review and assessment of data quality
Source: Water Res. 2019 May 15;155:410–22. doi: 10.1016/j.watres.2019.02.054 (PMC6449537; doi:10.1016/j.watres.2019.02.054)
Supplement: Multimedia component 1 [file mmc1.docx]

**Supporting Information**

**Microplastics in Freshwaters and Drinking Water: Critical Review and Assessment of Data Quality**

Albert A. Koelmans^†^, Nur Hazimah Mohamed Nor^†^, Enya Hermsen^†^, Merel Kooi^†^, Svenja M. Mintenig^‡,§^, Jennifer De France^ǂ^

^†^Aquatic Ecology and Water Quality Management Group, Wageningen University, The Netherlands.

^‡^Copernicus Institute of Sustainable Development, Utrecht University, The Netherlands.

^§^KWR Watercycle Research Institute, Nieuwegein, The Netherlands.

^ǂ^ World Health Organisation (WHO), Avenue Appia 20, 1211 Geneva, Switzerland

**Table S1:** Study Characteristics

| **Reference** | **Country** | **Source** | **Treatment** | **Sampling Date** | **Size, shape** | **Polymers, chemicals** | **Value** | **Detection limit, negative and positive controls and blanks.** | **Sampling method** | **Analysis method** | **Comments** |
| --- | --- | --- | --- | --- | --- | --- | --- | --- | --- | --- | --- |
| **Anderson et al. 2017** | Lake Winnipeg, Canada | LAK | n.a. | Jul 2014 - Jun 2016 | Fibres, fragments, film and foam (pellets not found). | n.i. | Mean: 193 420 (±115 567 SD) #/km^2^  Range: 52 508 – 748 027 #/km^2^ | DI water blanks (quadruplicates, 480L). Air blanks for 24h. Corrected for both blanks. | Manta trawl; 333 μm mesh. Preserved in 70% ethanol. | Samples rinsed and large objects removed; WPO treatment with Fe(II), heated to 75 °C; visual inspection of subsamples for plastics; subset of particles identified with SEM-EDS. |  |
| **Baldwin et al., 2016** | Great Lake tributaries, US | RIV | n.a. | Apr 2014 - Apr 2015 | Size: 0.355-0.999, 1.00-4.759, ≥4.75 mm;  Shape: Fragments, pellets/beads, lines/fibers, films and foam. | n.i. | Mean: 4.2 x 10^-3^ #/L;  Median: 1.9 x 10^-3^ #/”L;  Range: 0.05 - 32 (x10^-3^) #/L | Five negative controls in the field and 11 in the lab were included. | Neuston net (333 µm). Sample volume measured. . Net rinsed with tap water or filtered (333 µm) stream water. Mesh cod content transferred to glass jars with spoon and tap water. Preserved in isopropyl alcohol. | Sieving through 4.75, 1.00 and 0.355 mm mesh. WPO with Fe(II) catalyst at 75°C). WPO solution sieved through 125 µm and MP visually identified under dissection microscope (40x). |  |
| **Browne et al. 2011** | West Hornsby and Hornsby Heights, NSW, AU | WWTP | 3° treatment | 2010 | n.i. | PEST, PMMA and PA | EF mean: 1 #/L | n.i. | Samples collected in glass bottles with metal caps. | Filtered and identified with Transmittance FT-IR |  |
| **Cable et al. 2017** | Lakes Superior, Huron, Eerie and St. Clair, USA | LAK | n.a. | May - Aug 2014 | Size: >4750, 4750-1000 and 106-1000 µm.  Shape:  For > 1000 µm: fragment, film, foam, line, nurdle, sphere, paint or fibre.  For 106-1000 µm: fragment or fibre. | n.i. | Mean: 465 606 (± 403 378) #/km^2^ (106-1000μm); 32 219 (± 73 576) #/km^2^ (1000-4750 μm); 3 503 (± 12 766) #/km^2^ (>4750 μm).  Range: 126 933 – 1 910 562 #/km^2^ | n.i. Negative controls (n = 3) included. | Manta trawl; 100 μm mesh. Triplicate trawls, for 20 min. | All size classes: 10% sodium dodecyl sulphate at 50 °C, size fractioned. 106-1000 µm: incubated with proteinase, cellulase, and chitinase, followed by incubation with 30% H_2_O_2_, followed by WPO treatment. Visual sorting with stereo dissecting microscope. Small subset analysed with SEM-EDS. | Focus on avoiding contamination, including SEM-EDS to generate library of signatures of potential confusing items, and of suspected plastic and suspected non-plastic particles |
| **Carr et al. 2016** | Los Angeles, US | WWTP | 2° & 3° | June 2014 - Jan 2015 | Size: (20), 45, 180, 400 µm.  Shape: spheres, fragments and fibres. | n.i. | (1) Skimming Tertiary EF: 3-23 MP in 9.46-9.57 × 10^6^ L skimmed; (2) Secondary EF: 1 MP in 5.68 × 10^4^ L; (3) Final EF: 0 MP in 1.89 × 10^5^ L | n.i. | Method 1: EF sieved through stacked stainless steel sieves (400, 180, 45 and only 2 events used 20 µm). Flows-11.4-22.7 L min^-1^.  Method 2: Skimmed final effluent outfall with surface filtering assembly. Collected sample until clogging. | Tertiary EF: Centrifuging at 4000 RPM for 20 min.  Secondary EF: subsamples of 5mL in gridded petri dish, 20% of total sample. Skimming: digestion with bleach.  All samples were examined under microscope and checked with a micro-spatula. Some MPs analysed with ATR- FTIR. |  |
| **Di et al. 2018** | Three Gorges Reservoir, CN | RIV | n.a. | Aug 2016 | Size: <0.5, 0.5-1, 1-2, 2-3, 3-4 mm;    Shape: Fibre, fragment, pellet, film and styrofoam. | PS, PP, PE, PC, PVC, VC/VAC;  Nonanoic acid, 4-aminobenzoic acid, p-tolualdehyde, pth-methionine | Mean (s.d.): 4.703 (± 2.816) # /L; Range: 1.597-12.611 #/L | n.i. | Pumped 25L of water from 1m depth (2 reps) and filtered through 48 μm sieve. contents washed into jar using pure water, samples fixed in 5% formalin and stored at 4°C. | Digestion with H_2_O_2_^.^ Solution filtered through 0.45 µm and dried at 50°C. MP visually inspected under a dissecting microscope. Subset analyzed with micro-Raman spectroscopy and SEM. |  |
| **Dris et al. 2015** | Seine-Centre WWTP, Paris, France | WWTP | 2° treatment | 8-10 April 2014 | Size: 100-500 µm, 500-1000 µm, 1000-5000 µm.  Shape: Fibre | n.i. | IF: mean 293 (range: 260-320) #/L  EF: mean 35(range: 14-50) #/L | Blanks included, # fibres negligible. | Collected with automatic sampler and 24-h average samples analysed. A 0.05L aliquot was analysed. | Samples filtered on filter (1.6 µm) and particles counted with stereomicroscope (16x). |  |
| **Dris et al., 2015** | River Seine, River Marne, Paris, France. | RIV | n.a. | 26 June, 17 July, 3 December 2014 | Size: 100-500 µm, 500-1000 µm, 1000-5000 µm.  Shape: Fibre | n.i. | Plankton net: mean 30 (range 3 – 106) 10^-3^ #/L. Manta trawl: mean 0.35 (range 0.28 – 0.45) 10^-3^ #/L. | Blanks included, # fibres negligible. | Plankton net (80 µm mesh) for 1 min. Manta trawl (330 µm mesh) for 15 min. | Samples filtered on filter (1.6 µm) and particles counted with stereomicroscope (16x). |  |
| **Dris et al. 2018** | Seine River, Marne River, Paris, France | RIV | n.a. | Apr 2014 – Dec 2015 | Size: 50 – 5000 µm. | PET, PP, PA, PET-PUR (and cellulosic fibres). | Concentration means and ranges at 5 sites (10^-3^ # / L): 100.6 (5.7-398.0), 48.5 (2.7-441.4), 27.9 (3.2-92.2), 27.9 (2.4-156.6), 22.1 (1.0-85.0). | Blanks included, # fibres negligible. | Plankton net with mesh size 80 μm; triplicate sampling under bridges for 1 min. | Digestion with SDS, biozyme and H2O2 (Mintenig et al. 2014); Density separation with ZnCl_2_ (>1.6 g cm^-3^ ). Sorting with stereomicroscope. Small subset of fibres checked with micro-FTIR spectroscopy. |  |
| **Dyachenko et al. 2017** | East Bay Municipal Utility District, California, US | WWTP | 2° treatment | n.i. | Size: 5-1 mm, 0.355 - 1mm, 0.125-0.355 mm;  Shape: Fibre, film, foam, fragment, pellet. | Polyacrylic, PP, PE | Max:  24-hour sampling- 0.02 #/L;  2-hour sampling- 0.17 #/L. | P.C. for PS, 87% recovery, no replicates. | Effluent flow filtered through 5, 1, 0.355 and 0.125 mm stacked sieves. Flow of 1 gal/min for 24 hours. or 2-hour composites at peak flow. Sieve contents transferred with DI water into glass jars and stored at 4°C. | WPO with FeSO_4_ catalyst at 70°C. WPO solution filtered through 0.8 µm. Examined with dissecting microscope (45X). Micro- FTIR for most commonly observed particles. | No concentrations mentioned, no volumes, random particle identification |
| **Eriksen et al., 2013** | Laurentian Great Lakes, US | LAK | n.a. | 11 - 31 Jul 2012 | Size: 0.355-0.999, 1.00-4.759, ≥4.75 mm;  Shape: Fragment, pellet, line, film and foamed PS. | n.i. | Mean: 43157 #/km^2^;  Range: 0 - 466305 #/km^2^. | n.i. | Manta trawls (333 µm) deployed for 60 min. Tow speed noted. Preserved in 70% isopropyl alcohol. | Samples rinsed in salt water and sieved through 4.75, 1.00 0.355 mm mesh. <1mm particles analysed with SEM. Plastic sorted under a dissecting microscope. |  |
| **Estahbanati et al. 2016** | Raritan River, New Jersey, USA | RIV | n.a. | Oct - Nov 2015 | Size: 63 – 125, 125 – 250, 250 – 500, 500 – 2000 µm. | n.i. | Mean for 125-2000 µm: upstream WWTP: 24 (±11.4) 10^-3^ #/L; downstream WWTP: 71.7 (± 60.2) 10^-3^ #/L. | N.C. DI water over plankton net, values not included. P.C. spiked PE over plankton net, recoveries reported. | Plankton nets (mesh size 153 µm) deployed for 1h. | Nets rinsed with DI 3x. Sieves 4000, 2000, 500, 250, 125 and 63 µm. Particles > 2000µm discarded. Dried at 90°C, WPO with Fe(II) digestion at 75°C. Density separation with sodium chloride (density unknown). Visual inspection with reflected microscope. At least ¼ counted. | . |
| **Faure et al. 2015** | Lakes Geneva, Constance, Neuchâtel, Maggiore, Zurich, and Brienz, Switzerland. River Rhone, Aubonne, Venoge, Vuachère. | LAK, RIV | n.a. | Jul – Oct 2013 | Size: 300 – 5000 µm.  Shape:Fragments, pellets, beads, lines, fibres, films, foams. | Polymers: PE, PP, PS;  Contaminants: PCBs, OCPs, PAHs, PBDEs, BPA, nonylphenol, and phthalates | Lakes: Mean 91 000 (±120 000) #/km-2 or 26 000 (33 000) mg/km2.  Median: 48 000 #/km^2^ or 8 500 mg/km2.  Rivers: Mean 7.0 (± 0.20) 10^-3^ #/L.  Median: 0.36 10^-3^ #/L | n.i. | Manta trawl; 300μm mesh. Mean volume: 360 m^3^. | Plastics were visually detected in samples with stereomicroscope; 375 (all 169 macroplastic, 206 (10% of total) of microplastics) were analysed with ATR FTIR; the same samples were used for chemical analysis to determine pollutants. | Also included macroplastic (> 5000 µm). |
| **Fischer et al. 2016** | Lake Bolsena and Lake Chiusi, Apennines, IT | LAK | n.a. | 18-27 Aug 2014 | Size: 0.3-0.5, 0.5-1.0 and 1.0 - 5.0 mm;  Shape: Fragments/spherules and fibres. | n.i. | Mean: 2.49 x 10^-3^ #/L;  Range: 0.82-4.41 x 10^-3^ #/L | n.i. | Manta trawl (300 μm) sampling for 60 min. Cod end contents transferred to glass bottle, preserved with ethanol and stored in cool, dark place. | Sieved with 1, 0.5 and 0.3 mm mesh sizes. Density separation with NaCl (1.2 g/cm3). Hot digestion with HCl at 70°C. Samples filtered and stained with Nile red. UV-microscope. Subset of fibres verified with SEM. |  |
| **Free et al. 2014** | Lake Hovsgol, MN | LAK | n.a. | 19-26 Jul 2013 | Size: 0.355-0.999, 1.0-4.749, >4.75 mm;  Shape: Fragment, foam, line/fibre, pellet and film. | n.i. | Mean: 20 264 #/km^2^;  Range: 997 – 44 435 #/km^2^ | n.i. | Manta trawl (333μm), for 60 minutes. Storage in 70% ethanol. | Sieved through 0.335, 1.0 and 4.75 mm mesh. WPO with Fe(II) catalyst. Density separation with salt (1.62 g/ml). Visual identification with light microscope. |  |
| **Hendrickson et al. 2018** | Western Lake Superior, US | LAK | n.a. | 15 Aug2016 – 5 Jul 2017 | Shape: Foam, bead/sphere, fragment, fibres, film | PVC, PP, PE, PET, CPE, PS, PDMSand dodecyl phthalate resin | Mean (s.d.): 37 000 (27 000) #/km^2^ (1 200 mg/km^2^); Range: 0 - 110 000 #/km^2^ (91 – 3 538 mg/km^2^) | D.L.: Three times the average dev. of method blanks (5 particles/100 mL). N.C. duplicated air and replicate method blanks. P.C. in duplicate. | Manta trawl (333 μm), with flowmeter. On-site sieving with 4 mm and 250 μm mesh. Contents <4 mm transferred to glass container with forceps and rinsing. Stored in cool, dark place. Considered ambient contamination. | Dried at 90°C. WPO with Fe^2+^ at 75°C. Density separation with 5 M NaCl. Supernatant filtered and dried at RT or 90°C. Microscopy identification (40x) by two people simultaneously. Hot needle test. 10% of sorted particles were analysed with Pyrolysis GC-MS. If particles were big enough ATR-FTIR analysis was conducted prior to Pyrolysis GC-MS. | Detailed QA/QC procedures and accounted for detection limit. Units not convertible.  Minimum concentrations reported in different units is not logical (0 #/km^2^ and 91 mg/km^2^). |
| **Hoellein et al. 2017** | North Shore Channel, Lake Michigan, Wilmette, IL, US | RIV | n.a. | 7 Aug 2014 | Shape: Foam, film, fibre, fragment, pellet | PP, PS, PE | Range: 3.36 - 6.42 (x10^-3^) #/L | N.C. included DI water, corrected for. | Neuston net (333 µm). 4 replicates in 2 net deployments. Contents from net stored in acid washed containers. | Sieved through 4.75 and 0.3 mm mesh. Samples dried for 72h at 60°C. WPO with Fe(II) at 75°C. Density separation (6M NaCl). Filtered supernatant (0.7 μm) and dried at 60°C. Visual inspection with dissecting microscope (sub-counted fibres), Rep. samples analysed with Pyrolysis-GCMS. | S.I. mentions ipstream: 2.8 (0.5) (x10^-3^) #/L |
| **Kosuth et al. 2018** | CU, EC, UK, FR, DE, IN, ID, IE, IT, LB, SK, CH, UG, US | TAP | 17 out of 159 samples : Filtered;  8 out of 159 samples: CL.;  134 out of 159 samples: Treatment not mentioned. | Jan-Apr, 2017 | Shape: Fibres, fragments, film | n.i. | Mean: 5.45 #/L, Range: 0-61 #/L | N.C. (n = 30) included and corrected for. | Ran tap for 1 min, then flushed 500 ml HDPE bottle 2x (with sample), then sampled 457-603 ml (partly volunteers) | Filtration through 2.5 µm. Filtrate filtered again. Rose Bengal staining and visual identification with dissection microscope. Durability test with micro spatula. |  |
| **Lahens et al. 2018** | Saigon river, VN | RIV | n.a. | Dec 2015 - Apr 2016 | Fibres (bulk)50-4850 μm. Fragments (net): > 300 µm. | PET, PE, PP, PP, PS, PA, PVC, PE-PP copolymer, PP-vistalon, acrylic, polyepoxy, polyester, PE-ethyl acrylate | Fibres: 172-519 #/L (bulk sample), Fragments: 0.01 - 0.223 #/L (net). | n.i. | Fibres: 300 mL bulk sampling using bucket. Fragments: 300 µm mesh size net for 60 s, combined with a flowmeter. Contents transferred into glass container. | SDS for 24 h at 70 °C, enzyme digestion for 48 h at 40°C,  H_2_O_2_ digestion for 48 h at 40°C. Density separation with ZnCl_2_ (1.6 g/cm^3^). Filtration (2.7 µm) and microscopic inspection with image analysis software.76 fibres (10%) and 57 fragments (15%) were analysed by ATR FTIR. | Macroplastic was assessed too but not included in this scoring |
| **Lares et al, 2018** | Launialanselkä  Basin, Lake Saimaa, FI | LAK | n.a. | 10^th^ Oct 2016 – 2^nd^ Jan 2017 | Size: <0.25mm, 0.25-5.0mm, >5.0mm  Shape: Particles, fibres. | PES, PE, PA, PP | Average: 0.3 ± 0.1 (S.E.) #/L | N.C. included | Grab sampled 18.5-30.0L water at a location 100 m away from WWTP effluent outlet with a 10-L stainless steel bucket and poured over 2 sieves (0.25 and 5.0 mm). | Samples dried at 75°C in oven for at least 40h until dryness. WPO with Fe(II) heated to 75°C. Samples were vacuum filtration with cellulose nitrate filter, porosity (0.8 µm) and glass fibre filters (1.5 µm) at the bottom. Filters dried for 24h at room temperature covered with aluminium foil. Samples examined under digital optical microscope and classified representative samples (1.3-1.4% of overall particles) under FITR/Raman. |  |
| **Lares et al. 2018** | Kenkäveronniemi, Lake Saimaa, Mikkeli, FI | WWTP | 3° treatment | 10 Oct 2016-2 Jan 2017 | Size: <0.25mm, 0.25-5.0mm, >5.0mm  Shape: Fibres and particles.  Surface: Dull | PE, PA, PP | Mean IF: 57.6 ± 12.4 (S.E.) #/L  Mean EF: 1.0 ± 0.4 (S.E.) #/L. | Blanks included. | Grab sampled 4.0-30.0 L of IF and EF with 10-L stainless steel bucket and poured over 2 sieves (0.25 and 5.0 mm). Residues transferred with DI water in beakers and sealed with aluminium foil and rubber band for transfer to lab. Stored at 4°C in the dark. | Samples dried at 75°C in oven for at least 40h until dryness. WPO heated to 75°C. IF samples treated with cellulase for 24h at 40°C with 160 rpm shaking. Samples were vacuum filtrated with cellulose nitrate filter, porosity (0.8 µm) and glass fibre filters (1.5 µm) at the bottom. Filters dried for 24h at room temperature covered with aluminium foil. Samples examined under digital optical microscope and classified rep samples (1.3-1.4% of overall particles) using micro- FITR/Raman spectroscopy. |  |
| **Leslie et al. 2017** | Amsterdam, Netherlands | Canal | n.a. | 2012-2013 | Size: 10-300 and 300-5000 µm;  Shape: Fibres, spheres and foils. | n.i. | Mean: 100 #/L, Range: 48-187 #/L | N.C. included and corrected for. | Grab sampling with 2 L pre-rinsed (MQ) glass jars. Precautions to prevent contamination in the field | Filtration (0.7 µm) of 50 g or 100 g subsample, and microscopic inspection. | Data table is peculiar. |
| **Leslie et al. 2017** | Heenvliet, Amstelveen, Horstermeer, Blaricum, Amsterdam West, Westpoort, Houtrust, Netherlands | WWTP | n.a. | 2012-2013 | Size: 300-5000 µm and <300 µm.  Shape: Fibres, spheres, foils. | n.i. | IF: 68-910 #/L (mean range)  EF= 51-81 #/L (mean range)  Median EF: 52 #/L. Range: 9-91#/L | N.C. included and corrected for (2 fibres/blank). | Samples collected in 2L glass jars and stored in dark until analysis. | Samples were homogenized and 100 g aliquots were extracted. Sodium chloride solution was added to sample to saturation point (1.2 kg L^-1^) before filtration. | Did not report sampling details and WWTP processes and facilities. |
| **Magnusson and Noren 2014** | Långeviksverket, Lysekil, Sweden | WWTP | Tertiary treatment | 2014 | Shape: Fibre, fragment and flake. | PE, PP, thermoset plastic based on aliphatic polyester resin. | IF=15.1 ± 0.89 (SE) #/L  EF= 8.25 ± 0.85 (SE) 10^-3^ #/L | n.i. | Used a Ruttner sampler for influent and filter holder with tube for effluent. Filter over 300 µm mesh to collect 2 L of IF water per sample (triplicate) and  1000 L of EF per sample (quadruplicate). | Identification with stereo microscope (50x) Suspect fibres were placed on an object glass and heated over the flame of an alcohol burner. Subset of particles were picked out for ATR- FTIR analysis. |  |
| **Mani et al. 2015** | Rhine river, Switzerland, France, Germany, Netherlands. | RIV | n.a. | Jun - Jul, 2014 | Size: 300 – 5000 µm;  Shape: Spherules, fragments, fibres, foam. | PS, PP, acrylate, PEST, PMMA and PVC | Mean 892,777 #/km^2^;  Range: 52 364 -3 931 062 #/km^2^ | N.C. included for part of process. | Manta net (300 µm) with flowmeter; sampled vol. 60–250 m^3^. Samples handled against the wind and stored in tap water-rinsed glass jars and 10% NaCl solution. | Sieved through 5, 1, 0.3 mm mesh. SDS for 24 h at 70°C, enzyme digestion for 3 d at 37 °C, H_2_O_2_ for 24h at 37°C. Density separation, filtration (300 µm), microscopic inspection and ATR FTIR on 118 particles. |  |
| **Mason et al. 2016** | USA | WWTP | 2° and 3° treatment | Sep 2013 – May 2015 | Size: 125-355, >355 µm;  Shape: Fragments, pellet, line/fibre, film and foam | n.i. | Mean: 0.05 #/L;  Range: 0.004-0.195 #/L;  95% CI: 0.050-0.024 #/L. | N.C. included (7), no particles found. | Pumped effluent through 0.355 mm and 0.125 mm (12-18 L/min, for 2-24 hours). Preservation in 70% isopropyl alcohol. | WPO with Fe (II) catalyst. Sieved through 0.125 mm and transferred to petri dish.  Microscopic inspection (40x). |  |
| **Mason et al., 2016b** | Lake Michigan, US | LAK |  | 17 Jun-20 Aug 2013 | Size: 0.355-0.999, 1.00-4.759, ≥4.75 mm;  Shape: Fragments, pellet, line/fiber, film and foam. | HDPE, LDPE, PP, copolymers | Mean: 17 276 #/km^2^;  95% C.I: 12 898-21 655 #/km^2^;  Range: 0 - 100 016 #/km^2^ | N.C. included (6), no particles found. | Manta trawl (333 µm) for 30 min. Distance noted. Preserved in 70% isopropyl alcohol. | Sieving through 4.75, 1.00 and 0.355 mm mesh. WPO with Fe(II) catalyst for <4.75 mm particles and filtered again. SEM/EDS analysis for 20% subsamples (0.355-0.999 mm). ATR FTIR analyses for 59% subsamples (>4.75 mm). |  |
| **Mason et al. 2018** | CN, US, BR, IN, ID, MX, LB, TH | BOT | n.a. | n.i. | Size: 6.5-100, >100 µm;  Shape: Fragment, film, fibre, foam, pellet | PP, nylon, PS, PE, PEST (polyester + polyethylene terephthalate), Azlon, polyacrylates, copolymers | Mean: 325 #/L (> 100 μm - 10.4 #/L, < 100μm - 315 #/L);  Range: 0-10390 #/L | N.C.:  > 100μm - 4.15 (0-14) #/L,  6.5-100 μm - 23.5 (7-47) #/L. D.L. size: 6.5 um. P.C.: included for particles < 100 µm. | 259 bottles, 11 brands,27 different lots, 19 locations, 9 countries. 2-3 lots/brand for 10 brands, while 1 brand only had 1 lot. 9/10 bottles/lot (500-600 mL bot vol.); 4/6 bottles/lot (0.750-2 L bot vol.). One glass bottled water lot and others plastic. All bottles had plastic bottle caps. | Processed under laminar flow hood. NR for 30 mins, filtration (1.5 µm).  > 100 μm particles: microscopic inspection, ATR FTIR on subsample. 6.5-100 μm particles: NR tagged with software (av. results by 2 researchers). Image analysis validated with positive controls. Workspace wiped, materials rinsed, glassware covered, lab blanks (processed blindly). | Particles <100 μm were acknowledged not to be spectroscopically confirmed to be microplastics, however particles were expected to be plastic or of some other anthropogenic origin. |
| **McCormick et al., 2014** | North Shore Channel, Chicago, IL, US | RIV | n.a. | 13 Sep 2013 | Size: 0.330-2 mm;  Shape: Fragment, pellet, foam and fiber. | n.i. | Mean (SE): Upstream WWTP- 1.94 (0.81) x 10^-3^ #/L, Downstream WWTP - 17.93 (11.05) x 10^-3^ #/L. | Negative controls (n = 4): 4.5 ± 1.2 (mean ± SE) fibers/sample | Neuston net (333 µm) with flow meter deployed for 20 min. Rinsing of net with unfiltered site water, stored in Nalgene containers at 4 °C. | Sieving through 2 mm and 330 um mesh. Dried at 75°C. WPO with Fe(II) catalyst at 75°C for 48 h. Density separation with NaCl. Microscopic inspection (15% subsample). |  |
| **McCormick et al. 2016** | NE Illinois, Central Illinois and NW Indiana, US | RIV | Cl, de-Cl, UV, SF | 10 Jul-13 Oct 2014 | Size: 0.330-4.75 mm;  Shape: Pellets, fibres, fragments, foam, film | PE, PP, PS, ethylene | Mean (SE): Upstream - 2.355 (0.375) x 10^-3^ #/L ; Downstream - 5.733(0.850) x 10^-3^ #/L;  Range: 0.48-11.22 x 10^-3^ #/L | N.C. (n=5).: 4.67 fibres/sample | Neuston net (333 μm) with flow measurement for 15-20 min. Rinsing of net with unfiltered site water and stored in 1L containers at 4 °C. | Sieving through 4.75 and 0.330 mm.  Dried at 75°C. WPO with Fe (II) at 75°C. Density separation with NaCl (6 M). Filtration (0.7 µm) and microscopic inspection (fibres: 36% subsample). Pyr-GCMS (n=8). |  |
| **Michielssen et al. 2016** | Detroit and Northfield, US | WWTP | 2°and 3°treatment | March 25, 2016; Oct 19, 2015; March 21, 2016 | Shape: Fragments, fibres, paint chips, micro-beads | n.i. | Detroit:  IF=133.0 ± 35.6 #/L  Final EF=5.9 SAL L^-1^;  Northfield:  Final effluent = 2.6 SAL L^-1^  AnMBR system  Final effluent = 0.5 SAL L^-1^ | N.C. 20L (n = 1, 1 fibre found, not corrected for). | Grab sample in plastic containers cleaned with DI and air dried. Stored at 4C. | Sieved (4.74, 0.85, 0.3, 0.106 and 0.02 mm). Stereo-microscope. | SAL = small anthropogenic litter. Notation slightly confusing (removal or concentration). |
| **Miller et al. 2017** | Hudson river, US | RIV | n.a. | n.i. | Fibres | PET, fluoro-polymer/Teflon, PP | Median: 0.98 #/L, minimum: 0.625 #/L | N.C. included, air and water. Corrected for air, water negligible. | Grab samples (3 L from top 8-18 cm), pre-rinsed buckets / jars | Filtered over 0.45μm, filters in metal petri dishes, visual inspection, controls included, micro FTIR analysis |  |
| **Mintenig et al. 2017** | DE | WWTP | 2° treatment (n=8), 3° treatment (n=4) | 22-29 April, 2014 | Size: <500, >500 µm;  Shape: Fibres | PE, PP, PA, PVC, PS, PUR, silicone, paint, SAN, PEST, PET, EVA, PVAL, ABS, PLA. | Range:  >500μm: 0 – 40 x 10^-3^ #/L;  < 500μm: 10 – 9000 x 10^-3^ #/L | N.C. included and corrected for. | Pumped with filtration (10 µm SS filter) and flowmeter, 10 cm below water surface with pre-rinsing. Filtration unit sealed and stored at 4 °C., | Enzymatic maceration, SDS at 70 °C for 24 h, enzymatic digestion at 40-50 °C up to 6 d. Sonication in MQ for 3 mins. Filtration (500 µm).  <500 µm: WPO at 50 °C for 24 h and chitinase at 37 °C for 48 h and repeat WPO. Density separation with ZnCl_2_ (1.6 g/cm^3^), filtered (0.2 µm) and dried at 40 °C. FTIR imaging analysis (25%).  >500μm: Microscopic inspection and ATR-FTIR analysis for all particles. 60 fibres/sample analysed with FTIR imaging. |  |
| **Mintenig et al. 2019** | Germany | GROUND | None (raw water) | 13-20 Aug 2014 | Size: 50 – 150 μm;  Shape: fragments | PEST, PVC, PA, EPOXY resin, PE (relates to raw and tap water) | Mean: 0.7 x 10^-3^ #/L; Range 0 – 7 × 10^-3^ #/L | Size d.l. > 20 μm.  N.C. included and corrected for. | Extracted from wells at > 30 m depth. Filtered over 3 μm steel cartridge filters in housings from SAN and PP, at a flow of 5 L/min, volume 300 – 1000 L. Filtration until clogging. Pre-rinsing with Milli-Q. Samples stored at 4°C. | 0.01 M HCl treatment to remove CaCO_3_  and Fe-precipitates, then Milli-Q and 30% ethanol. Then 24 h WPO (35%) at 40°C and filters dried at 40°C.  FTIR-imaging applied to 100% of the filter but for particles only. Fibres were not identified. |  |
| **Mintenig et al. 2019** | Germany | TAP | Filtration / aeration of groundwater | 13-20 Aug 2014 | Size: 50 – 150 μm;  Shape: fragments | PEST, PVC, PA, EPOXY resin, PE (relates to raw and tap water) | Mean: 0.7 x 10^-3^ #/L; Range 0 – 7 × 10^-3^ #/L | Size d.l. > 20 μm.  N.C. included and corrected for. | Per consumer household: sampled at the water meter and at the conventional tap. Filtered over 3 μm steel cartridge filters in housings from SAN and PP, at a flow of 10 L/min, volume 1200 – 1500 L. Pre-rinsing with Milli-Q. Samples stored at 4°C. | 0.01 M HCl treatment to remove CaCO_3_  and Fe-precipitates, then Milli-Q and 30% ethanol. Then 24 h WPO (35%) at 40°C and filters dried at 40°C.  FTIR-imaging applied to 100% of the filter but for particles only. Fibres were not identified. |  |
| **Murphy et al. 2016** | River Clyde, Glasgow, Scotland (UK) | WWTP | 3°treatment | n.i. | Size: 0.598 ± 0.089 mm.  Shape: Flakes, fibres, film, beads and foam. | PMMA, alkyd, PET, PA, polyaryl ether, PEST, PE, PP, PS, PUR, polvinylfluride, PS acrylic, PVA, PVC, PVE | Mean (#/L): (1) IF- 15.70 ± 5.23 (SD or SE?); (2) Grit and grease- 8.70 ± 1.56; (3) Primary EF- 3.40 ± 0.28; (4) final EF: 0.25 ± 0.04. (SD or SE?) | N.C. included, but not considered and insufficient reported. | Grab sampling with 10 L steel buckets and sieved with 65 µm mesh.  Vol. sampled: (1) IF- 30 L; (2) EF-50 L. | Vacuum filtration with 11 µm filter paper. Subset (4/24^th^) of each filter paper analysed for particle count. Subset polymer identification using micro-FTIR. |  |
| **Oßmann et al. 2018** | Bavaria, DE | BOT | n.a. | n.i. | Size: ≤1.5, 1.5-5, 5-10, >10 µm | PTFE, Poly(p-phenylenterephthalamid, PS, PP, PE, PET+Olefin, PS + Olefin, PET, PVC, PA, Poly(diallylisophthalat), polyester, styrene-butadiene-copolymer, tris(2,4-di-tert-butylphenyl)phosphite | PET: Mean 2649 ± 2857 #/L (single-use), 4889 ± 5432 #/L (reusable); Range 90 – 16634 #/L  Glass: Mean 6292 ± 10521 #/L; Range 813 – 35436 #/L | N.C. included (one blank per analysis block). 7 blanks in total, on average 384 +/- 468 particles/L found. P.C. and D.L. not mentioned. | 32 samples from 21 brands purchased in Bavarian food stores. 12 reusable PET bottles (both newish and frequently reused), 10 single-use PET bottles, 9 reusable glass bottles and 1 single-use glass bottle. Volume per sample: 0.5 – 1.0 L. Targeted small particles (≥ 1 µm). | Labels removed, bottles cleaned with detergent, rinsed with DI water and dried in laminar flow box. Sample mixed by inverting bottle and transferred to cleaned flask and added EDTA. Then SDS was added and 250 ml aliquot of the solution was filtered through Al coated PC 0.4 µm membrane filter. Funnel of filtration unit rinsed with ethanol to remove foam and then UP water. Filters immobilized with metal rings and microscope slide, then identified with micro-Raman spectroscopy (4.4% filter area). |  |
| **Pivokonsky et al. 2017** | CZ | DWTP | WTP1: Coagulation, flocculation, SF; WTP2: sedimentation, SF and GAC filtration; WTP3: Coagulation-flocculation, flotation, SF and GAC filtration | Nov 2017-Jan 2018 | Size: 1-5, 5-10, 10-50, 50-100, >100 µm;  Shape: Fibre, spherical, fragment | PET, PP, PE, PS, PAM, PAM, PBA, PVC, Bakelite, PMMA, PPTA, PTT, DEHP | Raw: Range 1473 ±34 – 3605 ± 497 #/L  Treated: Range 338 ±76 – 628 ±28 #/L | Triplicate negative controls for each set of samples (per sampling day) | 1 L sample stored in pre-cleaned borosilicate glass bottles at 4°C. | WPO treatment with Fe(II), heated to 75 °C; Filtered through 5 µm then 0.2 µm PTFE (SEM analysis) and Al_2_O_3_ (FTIR) membrane filters and dried at 30°C for 30 mins. SEM analysis performed on 3 x (3 x 8 mm cutout). Micro-FTIR spectroscopy performed on >10 µm particles. Micro-Raman spectroscopy performed on 1-10 µm particles. ID on 25% of filter. |  |
| **Rodrigues et al. 2018** | Antuã River, PT | RIV | n.a. | May, Oct, 2016 | Shape: Fragments, pellets, films, foam and fibres | PE, PP, PS, PET, PVA, EVA, PTFE, PMMA, PAE, SBR, cellulose acetate | Range: 0.005-0.0517 mg/L and 0.058-1.265 #/L | N.C. included but amount negligible. | Motor water pump with 0.055 mm mesh net, sampling for 5 min at surface and 5 min at bottom. | Sieves 5 and 0.055 mm, WPO (75°C for 10 min, + 15 h room temperature). Density separation with zinc chloride (density 1.6 g cm^-3^). Vacuum filtration. Dried at 40°C for 3-5 days. Subsample of particles analysed with ATR-FTIR. |  |
| **Schymanski et al. 2018** | DE | BOT | n.a. | n.i. | Size: 5-10μm, 10-20μm, 20-50μm, 50 - 100μm, > 100μm | PEST, PE, PP, PA, | Single-use plastic bottles. Mean: 14 ±14 #/L;  Range: 2 - 44 #/L.  Returnable plastic bottles. Mean: 118 ± 88 #/L;  Range: 28-241 #/L.  Glass bottles.  Mean: 50 ± 52 #/L;  Range: 4-156 #/L.  Beverage cartons.  Mean: 11 ± 8 #/Ll;  Range: 5-20 #/L | N.C. (n=18): 1-42 plastic particles, mean: 14 ± 13 | 700 - 1500 ml, total volume of bottle was always used. Replica's: 12 returnable plastic bottles, 10 single use plastic bottles, 3 beverage cartons and 9 glass bottles. | Filtration over pre-counted filter, rinsing with MQ. Analyses with Singel Particle Explorer, u-Raman spectroscopy (1μm smallest particle size). | . SD or SE unknown. |
| **Sighicelli et al. 2018** | Lake Iseo, Lake Maggiore, Lake Garda, IT | LAK | n.a. | Summer 2016 | Size: > 300μm;  Shape: Fragment, balls, filaments, sheets, pellets. | PE, PP, PS, EPS, PET, Polyurethane, PVC, PEST, Acrylonitrile-Butadiene-Styrene | Lake Iseo.  Mean: 40000 #/km^2^  Lake Maggiore. Mean: 39000 #/km^2^  Lake Garda. Mean: 25000 #/km^2^ | n.i. | 22 trawls, average 6 per lake, and 6-9 additional per lake. Manta trawl with 300μm mesh size and 60x20 opening. Mean of 240 m^3^ water. | Manual separation with stereomicroscope. Drying at 50°C, counting, weighing. ATR-FT-IR for 46% subset. |  |
| **Simon et al. 2018** | DK | WWTP | 2° treatment | n.i. | Size: Up to 600 µm | Acrylate, SAN, VAC-PMMA copolymer, PE, PP, PE-PP co polymer, PEST, PS, PUR, PVC, EVA, PA, PVA | Raw wastewater median:: 7216 #/L or 250 ug/L  Treated wastewater median: 54 #/L or 4.2 ug/L  Recovery efficiency=99.3% | N.C.: triplicate blanks, not accounted for. P.C.: triplicates, not corrected for recovery.  D.L. raw waste water: 3093 #/L or 89 µg/L. | Sampled with auto samplers.  Raw WW samples filtered on-site through 10 µm stainless steel meshes. | Raw wastewater was wet-sieved with SDS. Sample incubated with cellulase enzyme for 48 h at 40°C then WPO. Reactor was kept in an ice-bath and temperature maintained between 15 and 30°C. 2-6% of homogenized sample transferred on transmission/ reflectance window, all analysed with FTIR- imaging. |  |
| **Su et al. 2016** | Taihu Lake, CN | LAK | n.a. | Aug 2015 | Size: 5-100, 100 - 333, 333-1000, 1000-5000 μm;  Shape: Fibres, pellets, films and fragments. | Cellophane, PET, PEST, terephthalic acid, PP | Range:  Plankton net-0.01-6.8 x 10^6^ #/km^2^;  Bulk-3.4-25.8 #/L | N.C. included and accounted for. | Plankton net (333 μm) for 1-30 min < 0.3 m deep. 250 mL sample collected and preserved in methyl aldehyde in glass bottle. Bulk surface sample: steel sampler, 5 L pooled sample. | Filtration (net-100 µm, bulk-5 µm). WPO at 65°C for 72 h. Microscopic inspection.Subset (113/1805 particles) analysed with micro-FT-IR or SEM/EDS. |  |
| **Talvitie et al. 2015** | FI | WWTP | Bar screening, grit removal, pre-aeration, primary sedimentation, activated sludge treatment, secondary sedimentation and tertiary biological filtration | Oct- Dec 2012 | Size: 200, 100 and 20μm;  Shape: fibres and particles. | n.i. | IF  Mean fibres: 180 #/L  Mean particles: 430 #/L.  Primary sedimentation Mean fibres: 14.2 (± 0.7) #/L  Mean particles: 290.7 (±28.2 ) #/L  After secondary sedimentation Mean fibres: 12.8 (± 1.6) #/L  Mean particles: 68.6 (± 6.3) #/L  EF  Mean fibres: 4.9 (± 1.4) #/L  Mean particles: 8.6 (± 2.5) #/L | N.C. included (n=?), no plastics found. | Pump, flow rate of 1.0 ml/min. Transparent plastic tubes (60 mm diameter), with 200, 100 and 20μm nets plasticized between connectors of tubes. Sample size: 0.3 - 285L. | Stereomicroscope (x50), identified and counted. particles and fibres. Blanks processed simultaneously | SD or SE? |
| **Talvitie et al. 2017** | FI | WWTP | Coarse screening, grit removal, chemical treatment and primary sedimentation, active sludge method. | Sep 2015 | Size: 20-100μm, 100-300μm, > 300μm;  Shape: fibres, fragments, flakes, films and spheres. | PES, polyacryl, PE, PS, PP | EF (general):  Range: 0.006 – 0.651 #/L (for different days), or 1.7E6 - 1.4E8 #/day.  Grab sample:  Range IF: 380 (± 52.2) - 686.7 (±155.0)  Range after pre-treatment: 9.9 (± 1.0) - 14.2 (± 4.0)  Range after AS: 1.0 (± 0.6) - 2.0 (± 0.2)  Range EF:  0.7 (± 0.6) - 3.5 (± 1.3).  24-hour composite sample:  Range IF: 390.0-900  Range after pre-treatment: 4.1-23.8  Range after AS: 1.5-2.8, EF: 1.4-2.8, blank: 0.4-0.8. | N.C. (n = 3), numbers reported. | 1. Grab samples: three replicates, pumping through tubes with 300, 100 and 20μm filter mesh. Sampling volume 0.1 l - 1 m^3^. IF: beaker because of clogging filters. 2. 24-h composite sample - 15 min intervals over 24-h period, for 3 days in a week. Sampling volume: 0.1 L - 14.5 L. 3. Sequential sampling: 1-h interval samples for 24 hours, pooled per 3 hours with automated samplers. | Stereomicroscope (50x). Particles counted, categorized in shapes. FTIR for 3 EF samples. In total 752 particles, but 18% success rate. |  |
| **Talvitie et al. 2017b** | FI | WWTP | 3°: micro-screen filtration with disc filters, rapid sand filters, dissolved air flotation, membrane bioreactor. | Apr 2014 - Aug 2015 | Shape: Fragments, flakes, films and spheres | PES, PE, PP, PS, PU, PVC, PA, acrylamide, poly-acrylate, alkyd resin, polyphenylene oxides, ethylene vinyl acetates. | Range before treatment: 6.9 (± 1.0) - 0.5 (± 0.2) #/L,  Range after treatment: 0.3 (± 0.1) - 0.005 (± 0.004) #/L,. | N.C. (n=3) included, no plastics found. | Three replicates, filter over 300, 100 and 20μm sieves with pump. Also 24-h composite samples. Water volume: 0.4 - 1000L. | Visual inspection, followed by an analysis using FTIR imaging for all pre- sorted particles. Blanks included. | Mentioning of "small sample volumes", and how this leads to false zero results. |
| **The Danish Environmental Protection Agency, 2017** | DK | WWTP | n.i. | n.i. | Size IF, median: 50μm  Size EF, median: 51.5μm. | Nylon, PE, PE-PP copolymer, PP, and PVC. | IF:  Median: 5.9  Mean: 8.0mg/L.  EF:  Median: 0.016, Mean: 0.034mg/L.  IF:  Median: 86000, Mean: 127000#/L.  EF:  Median: 6400, Mean: 5800 #/L. | D.L. IF > 4ug/L, EF > 0.20ug/L, Sludge > 20ug/g. P.C. included, recovery rates mentioned. | IF: 3 times 24h auto sampler. 1L stored in glass jar. EF: 3 times, 10μm filters until clogging of 3 filters (0.5 - 108 L per filter). Sludge: 2 times, 1 kg. | IF: 1mL sodium dodecyl sulphate addition, then 500μm pre-sieved. Cellulose digesting enzyme to 200 mL subsample. Incubation for 48h at 40°C, hydrolysed with H_2_O_2_. Fractions sieved: > and < than 80μm. From sieves to water + SDS. Filtered over 10μm mesh. Filters in ethanol, sonicated, scraped. 5mL ethanol. EF: the 3 10μm filters were hydrolysed and oxidized like IF. All samples: Micro-FT-IR. | Very concise report. |
| **Vermaire et al. 2017** | Ottawa River, CA | RIV | n.a. | Summer 2016 | Shape: Microfibres, microbeads, unidentified fragments | n.i. | Bottle sample median: 0.1 #/L. Manta trawl mean 0.00135 #/L | N.C. (n = 11) included and values reported. | Bottle sampling: 100 L over 100μm nylon mesh, triplicate per location. Manta trawls: 100μm mesh, 84-181 m^3^ (mean: 128, sd 37 m^3^). | WPO at 80°C for 7h. 100μm filter, Leica stereomicroscope 40x. |  |
| **Vermaire et al. 2017** | Ottawa River, CA | WWTP | n.i. | Summer 2016 | Shape: Microfibers, microbeads, unidentified fragments | n.i. | Median EF: 0.07 #/L | N.C. (n = 11) included and values reported. | 100 L EF, triplicate. ISCO peristaltic pump, 100μm nylon mesh | WPO at 80°C for 7h. 100μm filter, Leica stereomicroscope 40x. | Lower concentration than surface water (see above) |
| **Wang et al. 2017** | Wuhan, CN | LAK, RIV | n.a. | April 2016 | Size: 50 - 500μm, 500-1000μm, 1000-2000μm, 2000-3000μm, 3000-4000μm and 4000-5000μm;  Shape: Fibre, granule, film and pellet | PET, PP, PE, Nylon, PS | Range: 1.660 ± 0.6391 – 8.925 ± 1.591 #/L. | N.C. for field- and lab work included. | 20L pumped over 50μm sieve, in duplicates. | WPO at room temperature, Visual sorting of particles, a subsample analysed with SEM and micro-FTIR spectroscopy (2 particles per location). | Very small sampling volume (20L) |
| **Wang et al. 2018** | Dongting Lake and Hong Lake, CN | LAK | n.a. | Sep 2017 | Size: 50 – 5000 µm;  Shape: Fibre, granule and film. | PE, PP, PS, PVC | Dongting Lake: Mean: 1.19 #/L (> 330 um). Range: 0.900–2.8 #/L (50 – 5000 um)  Hong Lake:  Mean: 2.28 #/L (> 330 um) Range: 1.25–4.65 #/L (50 – 5000 um). | N.C. (n = 3 per lake) included, number negligible. P.C. included, recovery reported. | 20 L of bulk surface water (0–20 cm in depth) collected in twice (10 L  per time) using a Teflon pump, filtered through a stainless steel sieve with mesh 50-μm. Residues rinsed into glass bottle with distilled water, preserved in 4% formalin. GPS coordinates. | H_2_O_2_ at room temperature for 48 h, filtered, microscopic inspection and analysis with micro- Raman spectroscopy, blanks and positive controls included. |  |
| **Xiong et al. 2018** | Lake Qinghai area, CN | LAK, RIV | n.a. | July 2016 | Shape: Sheet, fibre, fragment, foam | PP, PE, PS, PET | Range lake: 0.05-7.58 E5 particles/km^2^.  Range rivers: 0.03-0.31 E5 particles/km^2^ river. | N.C. included (n = ?), not mentioned if corrected for. | Trawl net, 0.112 mm mesh, volume from flow & net size | Sieved over 1mm, density separation with potassium formate, then WPO at 60°C overnight then GF/C filters, visual examination. Analysis was done with micro- Raman spectroscopy. When numbers of sorted particles were < 100 µm, all particles were analysed. For higher concentrations (> 100 particles) 10-15% of particles were analysed. |  |
| **Zhang et al. 2015** | Three Gorges Reservoir, CN | LAK | n.a. | 23 Sep 2014 | Size: 112-300 μm, 300-500 μm, 500 μm - 1.6 mm, and 1.6-5 mm;  Shape: Fragments, sheets, line, foam. | PE, PP, PS in the form of Styrofoam | Range main stream Yangtze: 3407.7 E3 - 13 617.5 E3 #/km^2^  Range estuarine areas of the tributaries: 192.5 E3 - 11 889.7 E3 #/km^2^ | n.i. | Trawl, 112 μm mesh and 500 ml PE collecting bottle, transferred into 1 L glass bottle. Debris remaining in the net was rinsed with river water into a beaker and transferred into the same glass bottle. All samples preserved with methyl aldehyde and stored at 4°C before analysis. | Samples passed through a 1.6 mm stainless steel sieve. Transferred into 1 L separating funnels. Materials retained on the sieve were examined by naked eye and suspected plastic debris picked out. Samples in the funnel were allowed to settle. Floating debris on the surface transferred to petri dishes, oven-dried at 60°C, and examined using a stereomicroscope, analysis with ATR FTIR. |  |
| **Zhang et al. 2017** | Xiangxi River, tributary of the Three Gorges Reservoir, CN | RIV | n.a. | April, July, Oct 2015, and Jan 2016. | Size: 0.112−0.5 mm, 0.5−1 mm, and 1−5 mm;  Shape: sheet, fragment, lines, and foam. | PE, PP ,and expanded polystyrene (PS) | 0.55 E5 - 342 E5 #/km^2^ | n.i. | Trawl, 112μm mesh, transferred into 1 L glass bottle. Net rinsed 3x with distilled water. All samples preserved with methyl aldehyde and stored at 4°C before analysis. | Samples sieved, 1 mm mesh stainless steel sieve. Visual inspection. Suspected microplastics transferred to petri dishes for examination. Sieved water was collected and transferred into 1 L separating funnel. Density seperation (potassium formate, 1.5 g/mL). Samples in the funnel were allowed to settle overnight, then high density materials discharged. Micro Raman spectroscopy on all suspected microplastic particles |  |
| **Ziajahromi et al. 2017** | AU | WWTP | WWTP A: 1° treatment; WWTP B: 2° with UV; WWTP C: 3° with Cl, UF, RO | Oct2015 | Size: 25-100, 100-190, 190-500, 500 µm;  Shape: Irregular, granular and fibre. | PET, nylon, PE, PP, PS,PVC | Effluent:  WWTP A- 1.5 #/L,;  WWTP B: 0.48 #/L.;  WWTP C- 0.28 #/L, (3° treatment),0.21 #/L. | N.C. In = ?) included, no plastics found. P.C. included for part of sampling. | Pumped 3 - 200 L through stacked sieves of 500, 190, 100 and 25 μm at max flow rate of 10 L/min. Mesh screens stored on petri dishes sealed in Al foil. | Rinsed from sieves with UP water, and concentrated to 100 mL by drying at 90°C. WPO at 60 °C and dried. Density separation with NaI (1.49 g/ml). Centrifugation for 5 min at 3500xg. Supernatant filtered over 25 μm mesh and stained with Rose-Bengal solution. Dried at 60 °C for 15 min and microscopic inspection, analysis with ATR FTIR. | Method check with PS particles and staining method check with PE and polyester fibres. |

* calculated from reported data

**Table S1 (continued): Legend**

| Abbreviation | Full name |
| --- | --- |
| **Source** | |
| LAK | Lake |
| RIV | River |
| BOT | Bottled water |
| TAP | Tap water |
| WWTP | Wastewater treatment plant |
| DWTP | Drinking water treatment plant |
| **Treatment** | |
| 2° | Secondary |
| 3° | Tertiary |
| SF | Sand filtration |
| MBR | Membrane bioreactor |
| RO | Reverse osmosis |
| DIF | Disinfection |
| CL | Chlorination |
| OZ | Ozone disinfection |
| MF | Membrane filtration |
| IE | Ion exchange |
| GAC | Granular activated carbon |
| **Polymer types** | |
| PE | Polyethylene |
| PEST | Polyester |
| PVC | Polyvinyl chloride |
| PET | Polyethylene Terepthalate |
| PMMA | Poly (methyl) methylacrylate  a.k.a. acrylic |
| PS | Polystyrene |
| PA | Polyamide |
| PP | Polypropylene |
| PC | Polycarbonate |
| VC | Vinyl chloride |
| VA | Vinyl acetate |
| CPE | Chlorinated PE |
| PDMS | Polydimethylsiloxane |
| PES | Polysulfone |
| PVA | Polyvinyl acetate |
| PU | Polyurethane |
| SBR | Styrene butadiene rubber |
| EVA | Ethylene vinyl acetate |
| PAM | Polyacrylamide |
| PBA | Polybutylacrylate |
| **Chemicals** | |
| DI | Distilled water |
| MQ | Milli-Q water |
| SDS | Sodium dodecyl sulfate |
| NR | Nile Red |
| UP | Ultra-pure water |
| DEHP | Di(2-ethylhecyl)phthalate |
| PPTA | p-phenylene terephthalate |
| PTT | Polytrimethylene terephthalate |
| EDTA | Ethylene diaminetetra acetic acid tetrasodium salt |
| **Others** | |
| n.i. | No information |
| n.a. | Not applicable |
| MP | Microplastic |
| RT | Room temperature |
| Rep. | Representative |
| Pyr-GCMS | Pyrolysis gas chromatography mass spectrometry |
| IF | Influent |
| EF | Effluent |
| SEM/EDS | Scanning electron microscope with an elemental detection system |
| WPO | Wet peroxide oxidation with 30% H_2_O_2_ |
| SE | Standard error |
| SD | Standard deviation |
| SS | Stainless steel |

Abbreviations for countries: <http://www.realifewebdesigns.com/web-marketing/abbreviations-countries.asp>

Plastic compatibility with chemicals: <http://sevierlab.vet.cornell.edu/resources/Chemical-Resistance-Chart-Detail.pdf>

**Table S2**. Criteria used for the quantitative evaluation of the quality of microplastic concentration data.

| **Scores** |  |  |  |  |  |
| --- | --- | --- | --- | --- | --- |
|  |  |  | **2** | **1** | **0** |
| **Sampling** | **1** | **Sampling methods** | **Surface & Ground water:**   - Pump - Location - Materials used - Date - Depth of sampling   **Tap water:**   - Running tap before sampling - Flowrate - Source of tap water (tank/etc.) - Characteristics of sample   **Drinking water bottle:**   - Batch production lot - Flushing bottle 3 times with clean water - Shaking sample - Sparkling or still water   **WWTP/DWTP:**   - Location - Treatment - Date - Sampling method - Materials used   **No flushing with sample.** | The study reported only a subset of the required characteristics (e.g., date, location, materials used), however is still fairly reproducible. | No/ insufficient reportage of sampling methods. |
|  | **2** | **Sample size** | **Surface & ground water:** > 500 L  **Tap water/DWTP:** ≥1000L  **Drinking water bottle:** ≥10L per study unit (production batch) or n≥10 bottles  **WWTP:**   - Influent: 1L - Effluent: >500 L or until sieve clogging   *Sample volume may be smaller if target microplastic sizes are smaller* | **Surface water:** < 500 L “with good cause” (high concentrations e.g.)  Trawls without reporting volume is acceptable.  **Tap water/DWTP:** 10 – 1000 L  **Drinking water bottle**: 3<n<10 bottles  **WWTP:** If insufficient volume, sampling till clogging | **Surface water:** < 500 L  **Tap water/DWTP:** < 10L  **Drinking water bottle:** < 10L per study unit  **WWTP:** Insufficient sampling volume. |
|  | **3** | **Sample processing and storage** | Sample storing shortly after sampling; any sample handling was avoided before arriving in the laboratory. Sample containers should be rinsed with filtered water.  Sample preservation with chemicals should be justified and evaluated for compatibility.  *Manta trawl nets are allowed to be rinsed with unfiltered water.*  *Sieving in the field is acceptable if sample volume is large. Precautions should be taken to prevent contamination.* | Standards only partially met or containers are pre-rinsed with samples.  Citizen science approach with validation | Samples are handled outside. Storage not mentioned.  Citizen science approach without validation |
| **Contamination mitigation** | **4** | **Laboratory preparation** | - Cotton lab coat or non-synthetic clothes - Equipment and lab surfaces wiped and rinsed | - Solely wiping laboratory surfaces and equipment or not wearing a lab coat **IF** negative samples were run in parallel and examined for contamination. | No precautions. |
|  | **5** | **Clean air conditions** | - Clean room or laminar flow cabinet | Mitigation of airborne contamination by carefully keeping samples closed as much as possible **IF** negative samples were run in parallel and examined for occurring contamination. | No regard of airborne contamination, or solely use of *fume hood.* |
|  | **6** | **Negative control** | Controls (in triplicate) treated and analysed in parallel to actual samples.  Sample concentrations need to be reported accounting for controls. | Insufficient form of a control, e.g. the filtration of air, or the sole examination of petri dishes/ soaked papers placed next to the samples. | No negative controls. |
| **Sample purification/ handling** | **7** | **Positive control** | Controls (triplicate) with an added amount of microplastic particles treated the alongside the samples, and for which the particle recovery rates are determined. | Insufficient form of a positive control (e.g. if only a part of the protocol is tested). | No positive controls. |
|  | **8** | **Sample treatment (only for surface water and WWTP samples)** | Digestion of complete sample using a protocol with KOH, WPO and/or enzymes. If another chemical was used, effects on different polymers should be tested before application.  All sample treatments need to be carried out below 50°C to prevent any damage to microplastics. | If proof is missing that polymers are not affected by protocol (e.g. heated KOH)  **OR** in case studies exclusively focus on the bigger microplastics by sieving the samples (mesh size ≥ 300µm).  If WPO is carried out without cooling. | No digestion of sample. |
| **Chemical analysis** | **9** | **Polymer identification** | Per study; analysis of all particles when numbers of pre- sorted particles are <100. For particle numbers >100, 50% should be identified, with a minimum of 100 particles.  Per sample; analysis of all particles up to a maximum of 50 particles per sample.  Per filter: ≥25% of the surface area. | Insufficient polymer identification, potentially resulting in an unrepresentative subsample.  Identification with SEM/EDX to distinguish polymer vs non-polymeric materials. | No polymer identification. |

**Table S3. Scoring of individual studies**

|  | Anderson et al. 2017 (Surface) |  |  |
| --- | --- | --- | --- |
| 1 | Sampling methods | Sampling method (manta trawl), location, materials, date, depth mentioned. | 2 |
| 2 | Sample size | Trawling for >500 m, volume not mentioned. | 1 |
| 3 | Sample processing and storage | Nets rinsed, collected material preserved in 70% ethanol until laboratory processing; no rinsing of containers | 1 |
| 4 | Lab preparation | Not mentioned | 0 |
| 5 | Clean air conditions | Not mentioned | 0 |
| 6 | Negative controls | Air (duplicate) and DI (quadruplicated) tested and corrected for. | 1 |
| 7 | Positive controls | Not mentioned | 0 |
| 8 | Sample treatment (surface water) | WPO with Fe(II), 75°C | 1 |
| 9 | Polymer ID | Visual inspection; small subset identified with SEM-EDS | 1 |
| Total |  |  | 7 |

|  | Baldwin et al., 2016 (Surface) |  |  |
| --- | --- | --- | --- |
| 1 | Sampling methods | Discharge, drainage area, materials, method (neuston net), location, date, depth | 2 |
| 2 | Sample size | Volume not mentioned, net used until clogging | 1 |
| 3 | Sample processing and storage | Storage in glass jars (not rinsed), preserved in isopropyl alcohol | 1 |
| 4 | Lab preparation | Lab coats, non-synthetic clothing, negative controls included, no cleaning or rinsing mentioned | 1 |
| 5 | Clean air conditions | Lab air filtration system, samples processed in fume hood, samples covered, negative controls included | 1 |
| 6 | Negative controls | Negative controls for both field (n = 5) and lab (n = 11) included | 2 |
| 7 | Positive controls | Not mentioned | 0 |
| 8 | Sample treatment (surface water) | H_2_O_2_ digestion with Fe(II) catalyst (heated to 75°C) | 1 |
| 9 | Polymer ID | Not mentioned | 0 |
| Total |  |  | 9 |

|  | Browne et al, 2011 (WWTP) |  |  |
| --- | --- | --- | --- |
| 1 | Sampling methods | Only waste water treatment mentioned | 0 |
| 2 | Sample size | Sample size not reported | 0 |
| 3 | Sample processing and storage | Collection in pre-cleaned (unknown with what) glass bottles with metal caps | 1 |
| 4 | Lab preparation | Cotton clothing was worn. No other precautions mentioned | 0 |
| 5 | Clean air conditions | No regard of airborne contamination. | 0 |
| 6 | Negative controls | No negative controls. | 0 |
| 7 | Positive controls | No positive controls. | 0 |
| 8 | Sample treatment (surface water) | No digestion of sample. | 0 |
| 9 | Polymer ID | FTIR analysis of all particles but volume sampled may be under-representative of effluent. | 2 |
| Total |  |  | 3 |

|  | Cable et al. 2017 (Surface) |  |  |
| --- | --- | --- | --- |
| 1 | Sampling methods | Method (manta trawl 100 µm mesh), location, materials, date, season mentioned. | 2 |
| 2 | Sample size | Trawling for 20 minutes. Volume not mentioned. | 1 |
| 3 | Sample processing and storage | Cod-end rinsed over sieves, stored in plastic bottles in 70% ethanol (rinsing not mentioned) or in Ziploc bags when items were too big (rinsing not mentioned). | 1 |
| 4 | Lab preparation | Cotton lab coats; all liquid that contacted samples was filtered over 10 µm, glassware for storage was blasted with high pressure air; Teflon sheets inserted between glassware and their lids (no rinsing of surfaces mentioned) | 1 |
| 5 | Clean air conditions | Samples processed in laminar-flow or fume hood, otherwise covered. | 1 |
| 6 | Negative controls | Three negative controls of MQ processed simultaneously. Values reported, but not corrected. | 1 |
| 7 | Positive controls | Not mentioned | 0 |
| 8 | Sample treatment (surface water) | Sodium dodecyl sulphate (50 °C). Enzymes and WPO (75 °C). | 1 |
| 9 | Polymer ID | SEM-EDS on a subset of particles from smallest size class. | 1 |
| Total |  |  | 9 |

|  | Carr et al, 2016 (WWTP) |  |  |
| --- | --- | --- | --- |
| 1 | Sampling methods | Treatment mentioned. Pumped samples from plumbing and flows in plant facility. Dates reported. | 2 |
| 2 | Sample size | All volumes mentioned, and were sufficient. | 2 |
| 3 | Sample processing and storage | Samples stored in plastic centrifuge tube. No rinsing mentioned. | 1 |
| 4 | Lab preparation | No precautions. | 0 |
| 5 | Clean air conditions | Use of fume hood. | 0 |
| 6 | Negative controls | No negative controls. | 0 |
| 7 | Positive controls | No positive controls. | 0 |
| 8 | Sample treatment (surface water) | No digestion of samples | 0 |
| 9 | Polymer ID | Some particle analysed with ATR- FTIR | 1 |
| Total |  |  | 6 |

|  | Di & Wang 2018 (Surface) |  |  |
| --- | --- | --- | --- |
| 1 | Sampling methods | Pump, 1m depth, date, location reported. | 2 |
| 2 | Sample size | 25 L. | 0 |
| 3 | Sample processing and storage | Samples fixed in formalin and stored at 4°C | 2 |
| 4 | Lab preparation | Precautions taken and workplace cleaned, water and solutions used were filtered through 0.45 µm. | 2 |
| 5 | Clean air conditions | Not mentioned | 0 |
| 6 | Negative controls | Not mentioned | 0 |
| 7 | Positive controls | Conducted but no replicates. | 1 |
| 8 | Sample treatment (surface water) | Samples dried at 50%, WPO without catalyst | 2 |
| 9 | Polymer ID | Micro- Raman spectroscopy on 174 MPs, total count was unclear | 1 |
| Total |  |  | 10 |

|  | Dris et al, 2015 (WWTP) |  |  |
| --- | --- | --- | --- |
| 1 | Sampling methods | Materials, treatment, date mentioned. Method partly unclear (“automatic sampler and 24-averaged samples”) | 1 |
| 2 | Sample size | 0.05L | 0 |
| 3 | Sample processing and storage | Not mentioned | 0 |
| 4 | Lab preparation | Cotton laboratory coats, samples covered with tin foil, equipment heated at 500 °C. No rinsing of surfaces or materials mentioned. | 1 |
| 5 | Clean air conditions | Mitigation of airborne contamination by keeping samples closed with aluminium foil. Negative controls included. | 1 |
| 6 | Negative controls | Blanks included (n = unknown), number of fibres negligible | 1 |
| 7 | Positive controls | Not mentioned | 0 |
| 8 | Sample treatment (surface water) | Not mentioned | 0 |
| 9 | Polymer ID | Not mentioned | 0 |
| Total |  |  | 4 |

|  | Dris et al, 2015 (surface) |  |  |
| --- | --- | --- | --- |
| 1 | Sampling methods | Method (plankton net and manta trawl), materials, treatment, date mentioned. | 2 |
| 2 | Sample size | 450 – 2000 L. | 1 |
| 3 | Sample processing and storage | nets rinsed 3 times with river water into glass vessels (heated till 500 °C), covered with aluminium foil | 2 |
| 4 | Lab preparation | Cotton lab coats, samples covered with tin foil, equipment heated at 500 °C, cleaning of surfaces not mentioned | 1 |
| 5 | Clean air conditions | Mitigation of airborne contamination by keeping samples closed with aluminium foil. Negative controls included. | 1 |
| 6 | Negative controls | Blanks included (n = unknown), number of fibres negligible | 1 |
| 7 | Positive controls | Not mentioned | 0 |
| 8 | Sample treatment (surface water) | Not mentioned | 0 |
| 9 | Polymer ID | Not mentioned | 0 |
| Total |  |  | 8 |

|  | Dris et al. 2018 (Surface) |  |  |
| --- | --- | --- | --- |
| 1 | Sampling methods | Location, materials (plankton net with 80 µm mesh), dates, depth, flow mentioned. | 2 |
| 2 | Sample size | Triplicate of 1 min sampling, >2m^3^. | 2 |
|  |  |  |  |
| 3 | Sample processing and storage | Outside of net was rinsed with river water after collection. Storage not mentioned. | 0 |
| 4 | Lab preparation | Vessels and filters were heated to 500ºC; covered in aluminium foil at all times; cotton lab coats; filtration process could not be covered, but blanks were performed in this case. No rinsing of surfaces mentioned. | 1 |
| 5 | Clean air conditions | Not mentioned, but samples closed as much as possible and negative controls included. | 1 |
| 6 | Negative controls | Blanks included (n = unknown), number of fibres negligible. | 1 |
| 7 | Positive controls | Not mentioned | 0 |
| 8 | Sample treatment (surface water) | SDS at 70 °C, biozyme at 40 °C and WPO treatment at 40 °C. | 1 |
| 9 | Polymer ID | Small subset (25 fibres) identified with micro- FTIR spectroscopy. | 1 |
| Total |  |  | 9 |

|  | Dyachenko et al., 2017 (WWTP) |  |  |
| --- | --- | --- | --- |
| 1 | Sampling methods | Date not mentioned, materials, methods and treatments are mentioned. | 1 |
| 2 | Sample size | Not mentioned. | 0 |
| 3 | Sample processing and storage | Stored in glass jar at 4°C, rinsing not mentioned | 1 |
| 4 | Lab preparation | Not mentioned | 0 |
| 5 | Clean air conditions | Not mentioned | 0 |
| 6 | Negative controls | Not mentioned | 0 |
| 7 | Positive controls | Spiking with PS, 87% recovery. No replicates. | 1 |
| 8 | Sample treatment (surface water) | H_2_O_2_ with FeSO_4_, heated at 70°C | 1 |
| 9 | Polymer ID | Some samples identified with micro- FTIR, unclear how many | 1 |
| Total |  |  | 5 |

|  | Eriksen et al., 2013 (Surface) |  |  |
| --- | --- | --- | --- |
| 1 | Sampling methods | Date, method (manta trawl), location, tow speed and sea state using Beaufort scale mentioned. | 2 |
| 2 | Sample size | Volume not mentioned/calculated, 60 min trawling with manta net. | 1 |
| 3 | Sample processing and storage | Stored in isopropyl alcohol, container material unknown. | 1 |
| 4 | Lab preparation | Not mentioned | 0 |
| 5 | Clean air conditions | Not mentioned | 0 |
| 6 | Negative controls | Not mentioned | 0 |
| 7 | Positive controls | Not mentioned | 0 |
| 8 | Sample treatment (surface water) | No digestion of samples. | 0 |
| 9 | Polymer ID | Not mentioned | 0 |
| Total |  |  | 4 |

|  | Estahbanati et al. 2016 (Surface) |  |  |
| --- | --- | --- | --- |
| 1 | Sampling methods | Method (plankton net), location, materials, dates, depth mentioned | 2 |
| 2 | Sample size | >1 m^3^. | 2 |
| 3 | Sample processing and storage | Nets were transferred to lab for analysis. Storage not mentioned. | 1 |
| 4 | Lab preparation | Not mentioned | 0 |
| 5 | Clean air conditions | Not mentioned | 0 |
| 6 | Negative controls | DI water over plankton net, number unknown | 1 |
| 7 | Positive controls | Spiked PE over plankton net. Recoveries reported. (Duplicate, not triplicate) | 1 |
| 8 | Sample treatment (surface water) | Dried at 90ºC, WPO with Fe(II) heated to 75 °C. | 1 |
| 9 | Polymer ID | Not mentioned. | 0 |
| Total |  |  | 8 |

|  | Faure et al. 2015 (Surface) |  |  |
| --- | --- | --- | --- |
| 1 | Sampling methods | Method (manta trawl), materials mentioned. Dates unclear (“rivers after Oct 2013”). | 1 |
| 2 | Sample size | 320-430m^3^ | 2 |
| 3 | Sample processing and storage | Samples stored at 4 °C in polystyrene tubes in salt-saturated water until analysis (rinsing uknown). | 1 |
| 4 | Lab preparation | Clothes made of natural fibres, air exposure of samples limited, use of Milli-Q water, cleaning of tools and containers with stereomicroscope, work surface cleaning not mentioned. | 1 |
| 5 | Clean air conditions | Not mentioned | 0 |
| 6 | Negative controls | Not mentioned | 0 |
| 7 | Positive controls | Not mentioned | 0 |
| 8 | Sample treatment (surface water) | WPO with Fe(II) (Baker protocol, 75 °C). | 1 |
| 9 | Polymer ID | All macroplastics (n=169) and 10 % (n= 206) of sorted microplastics, randomly chosen, identified with ATR-FTIR. | 1 |
| Total |  |  | 7 |

|  | Fischer et al., 2016 (Surface) |  |  |
| --- | --- | --- | --- |
| 1 | Sampling methods | Date, lake characteristics, method (manta trawl), weather conditions mentioned | 2 |
| 2 | Sample size | Volume not mentioned explicitly | 1 |
| 3 | Sample processing and storage | Stored in glass bottles (rinsing not mentioned), ethanol, cool place. | 1 |
| 4 | Lab preparation | No precautions mentioned | 0 |
| 5 | Clean air conditions | Samples closed as much as possible but no negative controls were run | 0 |
| 6 | Negative controls | Not mentioned | 0 |
| 7 | Positive controls | Not mentioned | 0 |
| 8 | Sample treatment (surface water) | Hot digestion with HCl for 48h room temperature + 1h at 70°C | 1 |
| 9 | Polymer ID | No polymer ID mentioned | 0 |
| Total |  |  | 5 |

|  | Free et al., 2014 (Surface) |  |  |
| --- | --- | --- | --- |
| 1 | Sampling methods | Date, method (manta trawl), weather condition mentioned. | 2 |
| 2 | Sample size | Volume unknown, 60 min per trawl | 1 |
| 3 | Sample processing and storage | Storage in 70% ethanol, containers not mentioned | 1 |
| 4 | Lab preparation | Not mentioned.. | 0 |
| 5 | Clean air conditions | Not mentioned. | 0 |
| 6 | Negative controls | Not mentioned. | 0 |
| 7 | Positive controls | Not mentioned. | 0 |
| 8 | Sample treatment (surface water) | H_2_O_2_ digestion with Fe(II) catalyst. | 1 |
| 9 | Polymer ID | No polymer ID mentioned | 0 |
| Total |  |  | 5 |

|  | Hendrickson et al., 2018 (Surface) |  |  |
| --- | --- | --- | --- |
| 1 | Sampling methods | Location, method (manta trawl), materials used and date mentioned. | 2 |
| 2 | Sample size | Volume unknown, total surface area sampled:1.56E-2 km^2^. | 1 |
| 3 | Sample processing and storage | Collected in combusted glass containers with Teflon caps and stored in cool dark place | 2 |
| 4 | Lab preparation | Non-synthetic clothing, equipment rinsed, negative controls included (surface cleaning not mentioned) | 1 |
| 5 | Clean air conditions | Samples closed and negative controls were run during sampling and laboratory analysis. | 1 |
| 6 | Negative controls | Duplicate petri dishes left while sampling. Replicate method blanks performed (number of replicates unknown). | 1 |
| 7 | Positive controls | Included in duplicate for method testing. | 1 |
| 8 | Sample treatment (surface water) | Drying at 90°C, WPO with Fe^2+^ at 75°C | 1 |
| 9 | Polymer ID | 10% of sorted MP analysed with Pyrolysis GC-MS, and ATR-FTIR prior to Pyrolysis GC-MS if particles were big enough. | 1 |
| Total |  |  | 11 |

|  | Hoellein et al. 2017 (Surface) |  |  |
| --- | --- | --- | --- |
| 1 | Sampling methods | Method (neuston net), date, location, weather conditions | 2 |
| 2 | Sample size | Not mentioned. | 1 |
| 3 | Sample processing and storage | Acid-washed containers | 2 |
| 4 | Lab preparation | Covered with parafilm/Aluminium foil during sample processing. Accounted for procedural and reagent contamination. Cleaning and other precautions not mentioned. | 0 |
| 5 | Clean air conditions | Not mentioned. | 0 |
| 6 | Negative controls | Blanks with deionised water, corrected for in counts, number of blanks unclear | 1 |
| 7 | Positive controls | Not mentioned. | 0 |
| 8 | Sample treatment (surface water) | WPO with 0.05 M Fe(II) at 75°C | 1 |
| 9 | Polymer ID | Pyrolysis-GCMS on subset of sorted MP, but not clear how large | 1 |
| Total |  |  | 8 |

|  | Kosuth et al. 2018 (Tap) |  |  |
| --- | --- | --- | --- |
| 1 | Sampling methods | Method mentioned: tap run before sampling. Source not reported in detail. | 0 |
| 2 | Sample size | ~500 ml | 0 |
| 3 | Sample processing and storage | Not fully reported, partly done by volunteers / non-scientists, pre-rinsing with sample | 0 |
| 4 | Lab preparation | Cotton lab coats, lab surfaces and glassware cleaned & covered | 2 |
| 5 | Clean air conditions | Laminar airflow cabinet | 2 |
| 6 | Negative controls | Blanks included (n = 30) and reported, and background contamination accounted for | 2 |
| 7 | Positive controls | No positive controls | 0 |
| 8 | Sample treatment (surface water) | Not required for tap water | 2 |
| 9 | Polymer ID | No polymer identification performed for the water samples | 0 |
| Total |  |  | 8 |

|  | Lahens et al. 2018 (Surface) |  |  |
| --- | --- | --- | --- |
| 1 | Sampling methods | Location, date, materials, method (grab and trawl), season mentioned, depth and net type not mentioned. | 1 |
| 2 | Sample size | 0.3 L (fibres), or 60 s with net and flowmeter, then rinsed in glass container (fragments, unknown volume) | 1 |
| 3 | Sample processing and storage | Stored in glass container, rinsing not mentioned. | 1 |
| 4 | Lab preparation | Not mentioned. | 0 |
| 5 | Clean air conditions | Not mentioned. | 0 |
| 6 | Negative controls | Not mentioned. | 0 |
| 7 | Positive controls | Not mentioned. | 0 |
| 8 | Sample treatment (surface water) | SDS at 70°C, enzymatic and peroxide digestion at 40°C | 1 |
| 9 | Polymer ID | 76 fibres out of a total of 725, and 57 fragments of a total of 368, analysed with ATR-FTIR. | 1 |
| Total |  |  | 5 |

|  | Lares et al, 2018 (Surface) |  |  |
| --- | --- | --- | --- |
| 1 | Sampling methods | Method (Grab), materials, date, depth not mentioned | 1 |
| 2 | Sample size | Volume sampled 18.5-30L. Insufficient volume sampled and did not justify the cause for selecting volume. | 0 |
| 3 | Sample processing and storage | Samples sieved upon collection and transferred to laboratory in sealed beakers, storage details were provided, unclear if containers were rinsed. | 1 |
| 4 | Lab preparation | Filters and petri dishes were examined under microscope, surfaces wiped thrice with non-synthetic wipes, glass and metal dishes used. | 2 |
| 5 | Clean air conditions | Samples kept covered as much as possible. Negative samples run in parallel (from sampling in the field), and examined for occurring contamination. | 1 |
| 6 | Negative controls | Controls treated and analysed in parallel to actual samples. | 2 |
| 7 | Positive controls | No positive controls. | 0 |
| 8 | Sample treatment (surface water) | Samples dried at 75°C. H_2_O_2_ and Fe(II) digestion (75°C) | 1 |
| 9 | Polymer ID | Polymer identification (micro-FTIR and micro-Raman spectroscopy) on subsample(1.3-1.4%) of sorted particles and fibres. | 1 |
| Total |  |  | 9 |

|  | Lares et al, 2018 (WWTP) |  |  |
| --- | --- | --- | --- |
| 1 | Sampling methods | Method (Grab), materials, date, treatment mentioned. | 2 |
| 2 | Sample size | 4 - 30L | 0 |
| 3 | Sample processing and storage | Stored in sealed beakers (rinsing not mentioned) . | 1 |
| 4 | Lab preparation | Filters and petri dishes were examined under microscope, surfaces wiped thrice with non-synthetic wipes, glass and metal dishes used. | 2 |
| 5 | Clean air conditions | Samples kept covered as much as possible. Negative samples run in parallel (from sampling in the field), and examined for occurring contamination. | 1 |
| 6 | Negative controls | Controls treated and analysed in parallel to actual samples. | 2 |
| 7 | Positive controls | No positive controls. | 0 |
| 8 | Sample treatment (surface water) | Samples dried at 75°C. H_2_O_2_ and Fe(II) digestion (75°C) | 1 |
| 9 | Polymer ID | Polymer identification (micro-FTIR and micro-Raman spectroscopy) on subsample(1.3-1.4%) of sorted particles and fibres. | 1 |
| Total |  |  | 10 |

|  | Leslie et al, 2017 (Surface) |  |  |
| --- | --- | --- | --- |
| 1 | Sampling methods | Method (bulk sampling with glass jars), location and materials used mentioned. Date not mentioned. | 1 |
| 2 | Sample size | Samples were collected in 2 L glass jars; a 50 or 100 g subsample was analysed. | 0 |
| 3 | Sample processing and storage | Glass jars, pre-rinsed with MQ water | 2 |
| 4 | Lab preparation | Not mentioned. | 0 |
| 5 | Clean air conditions | No laminar flow hood, but procedural blanks included. | 1 |
| 6 | Negative controls | Procedural blanks, corrected for fibres, number of blanks unknown. | 1 |
| 7 | Positive controls | No positive controls. | 0 |
| 8 | Sample treatment (surface water) | No sample treatment. | 0 |
| 9 | Polymer ID | No polymer identification performed for the water samples. | 0 |
| Total |  |  | 5 |

|  | Leslie et al, 2017 (WWTP) |  |  |
| --- | --- | --- | --- |
| 1 | Sampling methods | Materials, methods known, date and treatment unknown | 1 |
| 2 | Sample size | Maximum sample size assumed to be 2 L since container volume size is 2 L. | 0 |
| 3 | Sample processing and storage | Stored in glass jars (pre-cleaned) | 2 |
| 4 | Lab preparation | Precautions were taken during sampling to avoid sample contamination the field. Precautions were taken in the laboratory by measuring blanks during analysis. No cleaning mentioned. | 0 |
| 5 | Clean air conditions | Mitigation of airborne contamination by analysing procedural blanks. | 1 |
| 6 | Negative controls | Controls treated and analysed in parallel to actual samples and reported a mean of 2 fibres per blank. Fibre concentrations reported were corrected for the blanks, number of controls unknown. | 1 |
| 7 | Positive controls | No positive controls. | 0 |
| 8 | Sample treatment (surface water) | No digestion of sample. | 0 |
| 9 | Polymer ID | Polymer identification performed only for sediment and biota samples from study and WW samples were assumed to have similar particles as the other components. | 0 |
| Total |  |  | 5 |

|  | Magnusson and Noren, 2014 (WWTP) |  |  |
| --- | --- | --- | --- |
| 1 | Sampling methods | Method (Ruttern sampler), materials, treatment and date mentioned. | 2 |
| 2 | Sample size | IF: 2L. EF: 1000 L. | 2 |
| 3 | Sample processing and storage | Samples were stored in petri dishes, no mention of rinsing | 1 |
| 4 | Lab preparation | Not mentioned | 0 |
| 5 | Clean air conditions | Not mentioned | 0 |
| 6 | Negative controls | Not mentioned | 0 |
| 7 | Positive controls | Not mentioned | 0 |
| 8 | Sample treatment (surface water) | Not mentioned | 0 |
| 9 | Polymer ID | ATR- FTIR analyses for small subset of sorted particles . | 1 |
| Total |  |  | 6 |

|  | Mani et al, 2015 (Surface) |  |  |
| --- | --- | --- | --- |
| 1 | Sampling methods | Method (manta trawl), location, materials, date. | 2 |
| 2 | Sample size | 60-250 m^3^ | 2 |
| 3 | Sample processing and storage | Bottles flushed with tap water | 1 |
| 4 | Lab preparation | Cotton coats, plastic/glassware rinsed and covered, cleaning of surfaces not mentioned. | 1 |
| 5 | Clean air conditions | No laminar flow hood, but blanks were run | 1 |
| 6 | Negative controls | Blanks were done for part of the process, not clear how many and if samples were corrected for blanks | 1 |
| 7 | Positive controls | Not mentioned. | 0 |
| 8 | Sample treatment (surface water) | SDS (70 °C), enzymes (37 °C), H_2_O_2_ (37 °C) | 1 |
| 9 | Polymer ID | ATR-FTIR on 118 out of 25 956 particles. | 1 |
| Total |  |  | 10 |

|  | Mason et al., 2016 (WWTP) |  |  |
| --- | --- | --- | --- |
| 1 | Sampling methods | Method (pump), flow-rate, date, location, treatments mentioned | 2 |
| 2 | Sample size | ≥500L | 2 |
| 3 | Sample processing and storage | Preserved in isopropyl alcohol, no mention of container rinsing. | 1 |
| 4 | Lab preparation | Not mentioned | 0 |
| 5 | Clean air conditions | Not mentioned | 0 |
| 6 | Negative controls | Blanks included (n=7), values recorded | 2 |
| 7 | Positive controls | No positive controls | 0 |
| 8 | Sample treatment (surface water) | WPO with Fe(II) (temperature not mentioned) | 1 |
| 9 | Polymer ID | No polymer identification | 0 |
| Total |  |  | 8 |

|  | Mason et al., 2016b (Surface) |  |  |
| --- | --- | --- | --- |
| 1 | Sampling methods | Method (manta trawl), date, location mentioned. | 2 |
| 2 | Sample size | Volume not mentioned/calculated, 30 min trawling. | 1 |
| 3 | Sample processing and storage | Stored in isopropyl alcohol, no mention of container rinsing. | 1 |
| 4 | Lab preparation | Not mentioned. | 0 |
| 5 | Clean air conditions | Not mentioned. | 0 |
| 6 | Negative controls | Six blanks included, no particles found. | 2 |
| 7 | Positive controls | Not mentioned. | 0 |
| 8 | Sample treatment (surface water) | WPO with Fe(II) catalyst (temperature not mentioned) | 1 |
| 9 | Polymer ID | Subset of >4.75 mm particles (59%) analysed with ATR-FTIR. Subset of particles 0.355- 0.999 µm (20%) analysed with SEM/EDS. | 1 |
| Total |  |  | 8 |

|  | Mason et al, 2018 (Bottle) |  |  |
| --- | --- | --- | --- |
| 1 | Sampling methods | Brand, lot, origin. No mention of flushing or shaking of bottles, polymer of bottle cap. | 1 |
| 2 | Sample size | Replicated bottles, total volume > 5-6 L | 2 |
| 3 | Sample processing and storage | Bottles opened in laminar flow hood | 2 |
| 4 | Lab preparation | Cotton lab coats, cleaning of lab and equipment not sufficient (once a week) | 1 |
| 5 | Clean air conditions | Laminar flow hood | 2 |
| 6 | Negative controls | Blanks included and fully reported | 2 |
| 7 | Positive controls | Positive controls included, but only for < 100 um particles | 1 |
| 8 | Sample treatment (surface water) | Not required for bottled water | 2 |
| 9 | Polymer ID | ATR-FTIR analysis on ~1000 particles (50%) of >100 um (not 50% of entire sample) | 1 |
| Total |  |  | 14 |

|  | McCormick et al., 2014 (Surface) |  |  |
| --- | --- | --- | --- |
| 1 | Sampling methods | Method (neuston net), date, location, materials mentioned. Depth not mentioned. | 1 |
| 2 | Sample size | Volume not mentioned, 20 min trawling | 1 |
| 3 | Sample processing and storage | Stored in Nalgene containers, no rinsing mentioned, at 4°C | 1 |
| 4 | Lab preparation | Not mentioned. | 0 |
| 5 | Clean air conditions | Not mentioned. | 0 |
| 6 | Negative controls | Four negative controls included, values corrected for the controls. | 2 |
| 7 | Positive controls | Not mentioned. | 0 |
| 8 | Sample treatment (surface water) | WPO with Fe(II) catalyst at 75°C | 1 |
| 9 | Polymer ID | Not mentioned. | 0 |
| Total |  |  | 6 |

|  | McCormick et al., 2016 (Surface) |  |  |
| --- | --- | --- | --- |
| 1 | Sampling methods | Method (neuston net) location, date mentioned. Depth not mentioned | 1 |
| 2 | Sample size | Trawl for 15-20 min, volume not mentioned. | 1 |
| 3 | Sample processing and storage | Stored in container (rinsing not mentioned) and at 4°C. | 1 |
| 4 | Lab preparation | Not mentioned. | 0 |
| 5 | Clean air conditions | Not mentioned. | 0 |
| 6 | Negative controls | Blanks included (n=5) and accounted for. | 2 |
| 7 | Positive controls | Not mentioned. | 0 |
| 8 | Sample treatment (surface water) | WPO with Fe (II) at 75°C | 1 |
| 9 | Polymer ID | Subset analysed (n = 8 particles) with Pyrolysis GC-MS. | 1 |
| Total |  |  | 7 |

|  | Michielssen et al, 2016 (WWTP) |  |  |
| --- | --- | --- | --- |
| 1 | Sampling methods | Method, treatment, date, location mentioned | 2 |
| 2 | Sample size | Sampling volumes met for influent (1-2 L) but insufficient volume sampled for effluent (34-38 L). | 1 |
| 3 | Sample processing and storage | Samples were stored in plastic containers (rinsed with DI water) at 4°C until analysis. | 2 |
| 4 | Lab preparation | Not mentioned | 0 |
| 5 | Clean air conditions | Not mentioned | 0 |
| 6 | Negative controls | A blank control sample was processed in parallel with samples. Blank was not accounted for as only 1 fibre was found. No triplicates were performed. | 1 |
| 7 | Positive controls | No positive controls. | 0 |
| 8 | Sample treatment (surface water) | No digestion of sample. | 0 |
| 9 | Polymer ID | No polymer identification. | 0 |
| Total |  |  | 6 |

|  | Miller et al, 2017 (Surface) |  |  |
| --- | --- | --- | --- |
| 1 | Sampling methods | Location, method (grab), materials, depth mentioned, no date mentioned | 1 |
| 2 | Sample size | 142 samples of 1 L | 0 |
| 3 | Sample processing and storage | rinsed glass jars, rinsed with tap water | 1 |
| 4 | Lab preparation | Pre-rinsed (with tap water) materials, cotton lab coats, cleaning of surfaces not mentioned | 1 |
| 5 | Clean air conditions | Triple rinsed and covered, no clean air | 1 |
| 6 | Negative controls | Many blanks, corrected for air blanks, not for water blanks (negligible) | 2 |
| 7 | Positive controls | not reported | 0 |
| 8 | Sample treatment (surface water) | No digestion | 0 |
| 9 | Polymer ID | 14 fibres were checked (14%) using micro- FTIR spectroscopy | 1 |
| Total |  |  | 7 |

|  | Mintenig et al., 2017 (WWTP) |  |  |
| --- | --- | --- | --- |
| 1 | Sampling methods | Method (pump), location, treatment, materials, date mentioned | 2 |
| 2 | Sample size | ≥390 L, but clogging reported | 2 |
| 3 | Sample processing and storage | Filtration units sealed and stored at 4 °C. | 2 |
| 4 | Lab preparation | Lab coats, rinsing of materials, negative controls, no mention of cleaning work surfaces | 1 |
| 5 | Clean air conditions | No laminar flow cabinet, but negative samples run in parallel | 1 |
| 6 | Negative controls | Triplicate negative controls. | 2 |
| 7 | Positive controls | Not mentioned. | 0 |
| 8 | Sample treatment (surface water) | Enzyme digestion + WPO, heating up to 70°C | 1 |
| 9 | Polymer ID | All sorted MP > 500 µm analysed with ATR- FTIR, for MP < 500 µm, 25% of filter surface analysed with FTIR imaging. | 2 |
| Total |  |  | 13 |

|  | Mintenig et al., 2019 (Tap) |  |  |
| --- | --- | --- | --- |
| 1 | Sampling methods | Date, location, method (pump), flow rate, running before sampling, source, characteristics | 2 |
| 2 | Sample size | 1200 – 2500 L | 2 |
| 3 | Sample processing and storage | Milli-Q rinsing, closed, kept at 4°C | 2 |
| 4 | Lab preparation | Cotton lab coats, non-synthetic fabric, lab surfaces wiped, equipment rinsed with milli-Q and covered | 2 |
| 5 | Clean air conditions | No clean room or laminar flow hood, however samples were kept close and blanks were run | 1 |
| 6 | Negative controls | Blanks (n=4) were included and samples were corrected for the mean | 2 |
| 7 | Positive controls | Not mentioned | 0 |
| 8 | Sample treatment (surface water) | 0.01 M HCl, H_2_O_2_ at 40°C | 2 |
| 9 | Polymer ID | All particles analysed, whole filter surface analysed with FTIR imaging. | 2 |
| Total |  |  | 15 |

|  | Mintenig et al., 2019 (Ground water) |  |  |
| --- | --- | --- | --- |
| 1 | Sampling methods | Date, location, method (pump), depth, flow rate, source, characteristics | 2 |
| 2 | Sample size | 300 – 1000 L or until sieve clogging | 1 |
| 3 | Sample processing and storage | Milli-Q rinsing, closed, kept at 4°C | 2 |
| 4 | Lab preparation | Cotton lab coats, non-synthetic fabric, lab surfaces wiped, equipment rinsed with milli-Q and covered | 2 |
| 5 | Clean air conditions | No clean room or laminar flow hood, however samples were kept close and blanks were run | 1 |
| 6 | Negative controls | Blanks (n=4) were included and samples were corrected for the mean | 2 |
| 7 | Positive controls | Not mentioned | 0 |
| 8 | Sample treatment (surface water) | 0.01 M HCl, H_2_O_2_ at 40°C | 2 |
| 9 | Polymer ID | All particles analysed, whole filter surface analysed with FTIR imaging. | 2 |
| Total |  |  | 14 |

|  | Murphy et al, 2016 (WWTP) |  |  |
| --- | --- | --- | --- |
| 1 | Sampling methods | Treatment, method, materials mentioned. Date not mentioned, location unclear. | 1 |
| 2 | Sample size | IF: 30 L  EF: Did not meet either > 500 L or clogging criteria. Study only filtered 50 L of EF before the sieves became clogged. | 1 |
| 3 | Sample processing and storage | Sieved on site, stored in glass bottles (Cleaned with distilled water) with distilled water, closed off. | 2 |
| 4 | Lab preparation | Cotton lab coats and natural fabric was worn at all times, surfaces wiped down and equipment cleaned and examined for MP contamination. | 2 |
| 5 | Clean air conditions | Monitoring MP contamination on lab benches via tape-lifting method and airborne particulates by atmospheric deposition on filters in petri dishes during sample processing. No clean air conditions. | 1 |
| 6 | Negative controls | Insufficient form of a control. Did not identify items found on filters and thus were improperly reported and negative controls were not considered in final results. | 1 |
| 7 | Positive controls | No positive controls. | 0 |
| 8 | Sample treatment (surface water) | No digestion of sample | 0 |
| 9 | Polymer ID | 4/24^th^ (16.6%) of filter analysed with micro-FTIR spectroscopy. | 1 |
| Total |  |  | 9 |

|  | Oßmann et al. 2018 (Bottle) |  |  |
| --- | --- | --- | --- |
| 1 | Sampling methods | Age of bottle, label material, usage type (single-use, reusable), carbonation reported, production batch not mentioned. | 1 |
| 2 | Sample size | 0.5 – 1.0 L is adequate for the smallest size fraction, but not for the larger size fraction that was also targeted. | 1 |
| 3 | Sample processing and storage | Exterior of bottles cleaned and dried in laminar flow box prior to transferring samples. | 2 |
| 4 | Lab preparation | Cotton lab coats and glassware rinsed with SDS, 50% ethanol and ultrapure water. Wiping of surfaces not mentioned but analysis was carried out in clean room. | 2 |
| 5 | Clean air conditions | Clean room and laminar flow box. | 2 |
| 6 | Negative controls | 7 blanks but did not mention if reported concentrations accounted for blanks. | 2 |
| 7 | Positive controls | No positive controls. | 0 |
| 8 | Sample treatment (surface water) | EDTA and SDS added to samples. 50% ethanol used to remove foam. | 2 |
| 9 | Polymer ID | 4.4 % filter area analysed with micro-Raman spectroscopy (particle sizes ≥ 1 µm) | 1 |
| Total |  |  | 13 |

|  | Pivokonsky et al., 2018 (raw & treated drinking water) |  |  |
| --- | --- | --- | --- |
| 1 | Sampling methods | Source of (surface) water, characteristics, date, treatments, materials and method mentioned. Location of DWTPs and their source waters not specified. | 1 |
| 2 | Sample size | 1 L is adequate for the smallest size fraction, but not for the larger size fraction that was also targeted. | 1 |
| 3 | Sample processing and storage | Samples stored in 1L pre-cleaned glass bottles at 4°C. | 2 |
| 4 | Lab preparation | Cotton clothing, equipment rinsed but wiping of surfaces not mentioned. | 1 |
| 5 | Clean air conditions | Lab air filtered with HEPA air filters. This doesn’t avoid sample contamination from cloths or synthetic particles that are already in the lab, however procedural blanks were included. | 1 |
| 6 | Negative controls | Triplicate negative controls each sampling day, contaminated with <5% of MP concentration in samples, so neglected. | 2 |
| 7 | Positive controls | No positive controls mentioned | 0 |
| 8 | Sample treatment (surface water) | WPO treatment with Fe(II) and heated to 75°C. | 1 |
| 9 | Polymer ID | Micro-FTIR (25% of filter surface, for >10 um) and micro-Raman imaging (25% of filter surface, for 1 - 10 um). Corrected MP numbers by percentages of non-plastic particles. | 2 |
| Total |  |  | 11 |

|  | Rodrigues et al., 2018 (Surface) |  |  |
| --- | --- | --- | --- |
| 1 | Sampling methods | Method (pump), materials, date, location, depth. | 2 |
| 2 | Sample size | 1.2m^3^ per site | 2 |
| 3 | Sample processing and storage | Stored in glass flasks (not rinsed) in fridge | 1 |
| 4 | Lab preparation | Not mentioned | 1 |
| 5 | Clean air conditions | Not mentioned | 0 |
| 6 | Negative controls | Negative controls included, but no procedural blanks. | 1 |
| 7 | Positive controls | Not mentioned. | 0 |
| 8 | Sample treatment (surface water) | H_2_O_2_ digestion at 75°C | 1 |
| 9 | Polymer ID | Subset analysed with ATR-FTIR, but unknown amount. | 1 |
| Total |  |  | 9 |

|  | Schymanski et al., 2018 (Bottle) |  |  |
| --- | --- | --- | --- |
| 1 | Sampling methods | Flushing with 50 mL, polymer of caps not mentioned for all bottles | 1 |
| 2 | Sample size | Per brand or batch, one bottle (750 - 1500 ml) | 0 |
| 3 | Sample processing and storage | Not relevant | 2 |
| 4 | Lab preparation | Very careful cleaning, including rinsing of exterior of bottles. | 2 |
| 5 | Clean air conditions | Laminar flow workbench | 2 |
| 6 | Negative controls | 18 replica's for negative controls, values reported | 2 |
| 7 | Positive controls | No positive controls | 0 |
| 8 | Sample treatment (surface water) | Not relevant | 2 |
| 9 | Polymer ID | All filters analysed using micro- Raman spectroscopy (Single Particle Explorer). | 2 |
| Total |  |  | 13 |

|  | Sighicelli et al., 2018 (Surface) |  |  |
| --- | --- | --- | --- |
| 1 | Sampling methods | Location, method (manta trawl), depth, season mentioned | 2 |
| 2 | Sample size | Mean 240 m^3^ | 2 |
| 3 | Sample processing and storage | Stored in glass vials (rinsing not mentioned), in H_2_O_2_ (30% at 4°C) in fridge | 1 |
| 4 | Lab preparation | Not mentioned | 0 |
| 5 | Clean air conditions | Not mentioned | 0 |
| 6 | Negative controls | No negative controls included | 0 |
| 7 | Positive controls | No positive controls included | 0 |
| 8 | Sample treatment (surface water) | Exclusively focussed on > 300 um, H_2_O_2_ (30% at 4°C) in fridge | 2 |
| 9 | Polymer ID | Total of 46% sorted particles analysed with ATR- FTIR. | 1 |
| Total |  |  | 8 |

|  | Simon et al, 2018 (WWTP) |  |  |
| --- | --- | --- | --- |
| 1 | Sampling methods | Collected sample with automatic sampler over 24 h. Raw wastewater filtered on site through 10 µm steel sieves. Treatment: S. Date not mentioned, locations not explicitly mentioned. | 1 |
| 2 | Sample size | Raw WW volume sampled was 1 L. Effluent sampled varied from 4.1-81.5 L. Volume standard for effluent was not met. | 1 |
| 3 | Sample processing and storage | Storage after sieving not explicitly mentioned. | 0 |
| 4 | Lab preparation | Precautions were taken such as minimizing plastic tools for sampling and analysis, muffling of steel filters, covering glassware with aluminium foils, no mention of cleaning surfaces. | 1 |
| 5 | Clean air conditions | Mitigation of airborne contamination by keeping samples closed with aluminium foil. | 1 |
| 6 | Negative controls | Blanks were run in triplicates and followed the same treatment as samples and accounted for in results. | 2 |
| 7 | Positive controls | Positive controls were performed with triplicate analysis. | 2 |
| 8 | Sample treatment (surface water) | Raw WW sample incubated with cellulase enzyme for 48 h at 40°C. Wet peroxide oxidation with Fe(II) and H_2_O_2_ was performed in ice-bath. | 2 |
| 9 | Polymer ID | 2-6% of homogenized sample transferred on transmission/ reflectance window, all analysed with FTIR- imaging. | 1 |
| Total |  |  | 11 |

|  | Su et al., 2016 (Surface) |  |  |
| --- | --- | --- | --- |
| 1 | Sampling methods | Method (plankton net and bulk surface), location, depth, date and season mentioned | 2 |
| 2 | Sample size | Plankton net (volume not explicitly mentioned) and 5L bulk sample. | 1 |
| 3 | Sample processing and storage | Samples stored in methyl aldehyde at 4°C, containers rinsed | 1 |
| 4 | Lab preparation | Lab coats, negative controls included, all equipment rinsed three times with filtered (0.45 um) tap water, cleaning of surfaces not mentioned. | 1 |
| 5 | Clean air conditions | Mitigation by keeping samples closed, negative controls included | 1 |
| 6 | Negative controls | Negative controls (number unknown) included and analysed | 1 |
| 7 | Positive controls | Not mentioned. | 0 |
| 8 | Sample treatment (surface water) | WPO at 65°C | 1 |
| 9 | Polymer ID | Subset of sorted particles (113 from the total 1805) analysed with micro-FTIR spectroscopy or SEM/EDS. | 1 |
| Total |  |  | 9 |

|  | Talvitie et al., 2015 (WWTP) |  |  |
| --- | --- | --- | --- |
| 1 | Sampling methods | Method (pump), materials, treatment, date mentioned | 2 |
| 2 | Sample size | 0.3 - 285 L, Volume < 1L for some samples | 1 |
| 3 | Sample processing and storage | Not mentioned | 0 |
| 4 | Lab preparation | Rinsing of equipment mentioned (tap water), clothing not mentioned, cleaning of work surfaces not mentioned | 0 |
| 5 | Clean air conditions | No clean air conditions | 0 |
| 6 | Negative controls | Number of blanks unknown , no contamination found | 1 |
| 7 | Positive controls | No positive controls | 0 |
| 8 | Sample treatment (surface water) | No sample treatment, particles ≥ 20 um | 0 |
| 9 | Polymer ID | No polymer identification | 0 |
| Total |  |  | 4 |

|  | Talvitie et al., 2017 (WWTP) |  |  |
| --- | --- | --- | --- |
| 1 | Sampling methods | Treatment, materials, methods, location, date mentioned | 2 |
| 2 | Sample size | Influent: 0.1L, effluent 2L (20 um sieve) | 0 |
| 3 | Sample processing and storage | Storage in clean petri dishes, all material rinsed with tap water | 1 |
| 4 | Lab preparation | Not mentioned. | 0 |
| 5 | Clean air conditions | Not mentioned | 0 |
| 6 | Negative controls | Triplicate negative controls | 2 |
| 7 | Positive controls | No positive controls | 0 |
| 8 | Sample treatment (surface water) | No sample treatment, particles ≥20 um included | 0 |
| 9 | Polymer ID | Subset of sorted particles (from 3 effluent samples) analysed with FTIR imaging. | 1 |
| Total |  |  | 6 |

|  | Talvitie et al., 2017b (WWTP) |  |  |
| --- | --- | --- | --- |
| 1 | Sampling methods | Method, date, materials, treatments described in detail | 2 |
| 2 | Sample size | Some sample volumes too small (0.4 L influent, 140 L effluent), but mentioned that this could result in false zero's. | 1 |
| 3 | Sample processing and storage | Storage in petri dishes or in container (rinsed with tap water), depending on sample method and stored in fridge | 1 |
| 4 | Lab preparation | Rinsing of equipment with tap water, negative controls included (tap water), no other precautions mentioned, no cleaning of surfaces mentioned. | 1 |
| 5 | Clean air conditions | Careful handling of samples, negative controls included | 1 |
| 6 | Negative controls | Triplicate negative controls included, no contamination found. | 2 |
| 7 | Positive controls | Not mentioned | 0 |
| 8 | Sample treatment (surface water) | Not mentioned | 0 |
| 9 | Polymer ID | All sorted particles and fibres analysed with FTIR- imaging. | 2 |
| Total |  |  | 10 |

|  | Vermaire et al., 2017 (Surface) |  |  |
| --- | --- | --- | --- |
| 1 | Sampling methods | Depth of sampling, materials, season, method mentioned | 2 |
| 2 | Sample size | Partly 100 L, partly 100 000 L (different methods) | 1 |
| 3 | Sample processing and storage | Manta net was backwashed with river water between samples, the cod-end was washed with deionized water. Packed in whirl-pak bag and stored in fridge | 2 |
| 4 | Lab preparation | Not mentioned | 0 |
| 5 | Clean air conditions | Not mentioned | 0 |
| 6 | Negative controls | 11 negative controls included: unfiltered tap water (from filtered source), values reported and corrected for | 2 |
| 7 | Positive controls | No positive controls included | 0 |
| 8 | Sample treatment (surface water) | H_2_O_2_ at 80°C | 1 |
| 9 | Polymer ID | Not included | 0 |
| Total |  |  | 8 |

|  | Vermaire et al., 2017 (WWTP) |  |  |
| --- | --- | --- | --- |
| 1 | Sampling methods | Depth and method mentioned. Treatments not mentioned | 1 |
| 2 | Sample size | 300 L | 0 |
| 3 | Sample processing and storage | Packed and stored in fridge (whirl-pak bag) | 2 |
| 4 | Lab preparation | Not mentioned | 0 |
| 5 | Clean air conditions | Not mentioned | 0 |
| 6 | Negative controls | 11 negative controls included: unfiltered tap water (from filtered source), values reported and corrected for | 2 |
| 7 | Positive controls | No positive controls included | 0 |
| 8 | Sample treatment (surface water) | H_2_O_2_ at 80°C | 1 |
| 9 | Polymer ID | Not included | 0 |
| Total |  |  | 6 |

|  | Vollertsen et al., 2017 (WWTP) |  |  |
| --- | --- | --- | --- |
| 1 | Sampling methods | Waste water treatment not mentioned, date not mentioned | 0 |
| 2 | Sample size | 1L influent, 3x clogging effluent (0.5 – 108 litres per filter) | 2 |
| 3 | Sample processing and storage | Raw waste water stored in glass jar. Treated WW was filtered on site over 3 filters of 10um, particles from filter were concentrated in 5 mL ethanol. Storage of treated WW not explicitly mentioned. | 1 |
| 4 | Lab preparation | Not mentioned | 0 |
| 5 | Clean air conditions | Not mentioned | 0 |
| 6 | Negative controls | Not mentioned | 0 |
| 7 | Positive controls | Spike raw waste water (recovery mentioned) | 2 |
| 8 | Sample treatment (surface water) | SDS + Enzyme digestion + H_2_O_2_ with unknown catalyst | 1 |
| 9 | Polymer ID | Micro- FTIR spectroscopy, however, analysed filter surfaces unknown | 1 |
| Total |  |  | 7 |

|  | Wang et al., 2017 (Surface) |  |  |
| --- | --- | --- | --- |
| 1 | Sampling methods | Method (pump), location, date mentioned, depth unknown | 1 |
| 2 | Sample size | 20 L | 0 |
| 3 | Sample processing and storage | glass jars (not rinsed), in formalin solution, in fridge | 1 |
| 4 | Lab preparation | Rinsing of materials three times with distilled water, covered with aluminium foil, stereomicroscopic check of petri dishes, lab coat, cleaning of workspace | 2 |
| 5 | Clean air conditions | Closed samples, negative controls included | 1 |
| 6 | Negative controls | Negative controls (triplicate) for field- and lab work included, accounted for | 2 |
| 7 | Positive controls | Not mentioned | 0 |
| 8 | Sample treatment (surface water) | H_2_O_2_ at room temperature | 2 |
| 9 | Polymer ID | Analysis for a subset of pre- sorted particles (2 particles per location) with SEM and micro- FTIR spectroscopy. | 1 |
| Total |  |  | 10 |

|  | Wang et al, 2018 (Surface) |  |  |
| --- | --- | --- | --- |
| 1 | Sampling methods | Date, location, depth, method (pump), materials | 2 |
| 2 | Sample size | 20 L, but concentrations high enough | 1 |
| 3 | Sample processing and storage | rinsing of filter with distilled water into glass bottle (unclear if rinsed), formalin storage | 1 |
| 4 | Lab preparation | cotton lab coat, gloves, rinsing and cleaning of equipment and surfaces | 2 |
| 5 | Clean air conditions | laminar flow hood | 2 |
| 6 | Negative controls | Field blank tests, plus lab procedural blanks (triplicate) | 2 |
| 7 | Positive controls | Included with 92.7% recovery | 2 |
| 8 | Sample treatment (surface water) | 30% H_2_O_2_. effects tested (no effect found) | 2 |
| 9 | Polymer ID | Raman on 50 particles per lake (the total is unclear) analysed with micro- Raman spectroscopy. | 1 |
| Total |  |  | 15 |

|  | Xiong et al, 2018 (Surface) |  |  |
| --- | --- | --- | --- |
| 1 | Sampling methods | Date, location, method (trawl), depth mentioned | 2 |
| 2 | Sample size | No volume reported, data expressed as #/km^2^. | 1 |
| 3 | Sample processing and storage | Stored in glass bottle (rinsing not mentioned) and preserved with 5% methyl aldehyde. | 0 |
| 4 | Lab preparation | Nitrile gloves, cotton lab coat, shower cap (plastic), covered container, desktop, hands, and clothes cleaned with sticky dust drum. | 1 |
| 5 | Clean air conditions | Fume hood, samples covered when not used, blanks included | 1 |
| 6 | Negative controls | Blanks included. Not indicated how many and if corrections for blanks were done | 1 |
| 7 | Positive controls | No positive controls | 0 |
| 8 | Sample treatment (surface water) | 30% H_2_O_2_, 60°C, overnight | 1 |
| 9 | Polymer ID | For samples with a low MP concentration (<100 particles all particles analysed, and 10-15% of particles analysed when sample concentrations were > 100 particles. Analysis done with micro- Raman spectroscopy. | 1 |
| Total |  |  | 8 |

|  | Zhang et al, 2015 (Surface) |  |  |
| --- | --- | --- | --- |
| 1 | Sampling methods | Location, trawl, , materials, date, depth mentioned | 2 |
| 2 | Sample size | Trawl, volume unclear | 1 |
| 3 | Sample processing and storage | Methyl aldehyde and stored at 4 C, no rinsing of containers | 0 |
| 4 | Lab preparation | No information provided | 0 |
| 5 | Clean air conditions | No information provided | 0 |
| 6 | Negative controls | No information provided | 0 |
| 7 | Positive controls | No information provided | 0 |
| 8 | Sample treatment (surface water) | no digestion | 0 |
| 9 | Polymer ID | 50 - 100 particles per site analysed with ATR-FTIR, but total MP numbers unknown. | 1 |
| Total |  |  | 4 |

|  | Zhang et al, 2017 (Surface) |  |  |
| --- | --- | --- | --- |
| 1 | Sampling methods | Method (surface trawling), location, date, materials | 2 |
| 2 | Sample size | Trawl, volume unclear | 1 |
| 3 | Sample processing and storage | Methyl aldehyde and stored at 4 °C, containers rinsed | 1 |
| 4 | Lab preparation | Cotton coat, containers washed and covered, cleaning work surfaces not mentioned. | 1 |
| 5 | Clean air conditions | Laminar flow hood | 2 |
| 6 | Negative controls | No information provided | 0 |
| 7 | Positive controls | No information provided | 0 |
| 8 | Sample treatment (surface water) | No information provided | 0 |
| 9 | Polymer ID | All presorted particles analysed with micro-Raman spectroscopy. | 2 |
| Total |  |  | 9 |

|  | Ziajahromi et al., 2017 (WWTP) |  |  |
| --- | --- | --- | --- |
| 1 | Sampling methods | Treatment, materials method (pump), date mentioned. | 2 |
| 2 | Sample size | Sample volume 3-200L for effluent (until clogging) | 2 |
| 3 | Sample processing and storage | Storage in clean petri dishes (rinsing not mentioned) and sealed in aluminium foil. | 1 |
| 4 | Lab preparation | Materials rinsed with ultra pure water, no wiping of surface, but negative controls included | 1 |
| 5 | Clean air conditions | Use of fume hood, but samples covered and negative controls included | 1 |
| 6 | Negative controls | Negative controls included, number of controls not mentioned. | 1 |
| 7 | Positive controls | Positive controls for sampling and analyses, only part of the process, number of controls unclear | 1 |
| 8 | Sample treatment (surface water) | Heating up to 90°C, WPO at 60 °C | 1 |
| 9 | Polymer ID | All pre-sorted particles analysed using ATR-FTIR. | 2 |
| Total |  |  | 12 |

**Table S4** Pairwise comparisons of microplastic number concentrations per water type, using Wilcoxon rank sum test, P value adjustment method: bonferroni. Statistically significant differences (p<0.05) are indicated in bold font.

|  | WWTP Influent | WWTP Effluent | Lake | River | Canal | Ground water | Untreated DWTP water | Treated Tap Water |
| --- | --- | --- | --- | --- | --- | --- | --- | --- |
| WWTP EF | **0.00085** | - |  |  |  |  |  |  |
| Lake | **<2E-16** | 1 |  |  |  |  |  |  |
| River | **<2E-16** | **<2E-16** | <2E-16 |  |  |  |  |  |
| Canal | **5.6E-10** | **1.6E-8** | 5.7E-8 | 1 |  |  |  |  |
| Groundwater | 1 | 1 | 1 | 1 | 1 |  |  |  |
| U. DWTP water | 1 | 1 | **0.01194** | **9.5E-5** | **0.00188** | 1 |  |  |
| T. Tap water | **<2E-16** | 1 | **1.3E-15** | **<2E-16** | **3.2E-8** | 1 | **0.00983** |  |
| Bottled wat. | **2.8E-6** | **6.2E-6** | **<2E-16** | **<2E-16** | **1.6E-14** | 1 | **0.04146** | **<2E-16** |

# References

1. Anderson, P., Warrack, S., Langen, V., Challis, J., Hanson, M., & Rennie, M. (2017). Microplastic contamination in Lake Winnipeg, Canada. *Environmental Pollution, 225*, 223-231.
2. Baldwin, A., Corsi, S., & Mason, S. (2016). Plastic Debris in 29 Great Lakes Tributaries: Relations to Watershed Attributes and Hydrology. *Environmental Science and Technology, 50*(19), 10377-10385.
3. Browne, M., Crump, P., Niven, S., Teuten, E., Tonkin, A., Galloway, T., & Thompson, R. (2011). Accumulation of microplastic on shorelines woldwide: Sources and sinks. *Environmental Science and Technology, 45*(21), 9175-9179.
4. Cable, R., Beletsky, D., Beletsky, R., Wigginton, K., Locke, B., & Duhaime, M. (2017). Distribution and Modeled Transport of Plastic Pollution in the Great Lakes, the World's Largest Freshwater Resource. *Frontiers in Environmental Science, 5*.
5. Carr, S., Liu, J., & Tesoro, A. (2016). Transport and fate of microplastic particles in wastewater treatment plants. *Water Research, 91*, 174-182.
6. Di, M., & Wang, J. (2018). Microplastics in surface waters and sediments of the Three Gorges Reservoir, China. *Science of the Total Environment, 616-617*, 1620-1627.
7. Dris, R., Gasperi, J., Rocher, V., & Tassin, B. (2018). Synthetic and non-synthetic anthropogenic fibers in a river under the impact of Paris Megacity: Sampling methodological aspects and flux estimations. *Science of the Total Environment, 618*, 157-164.
8. Dris, R., Gasperi, J., Rocher, V., Saad, M., Renault, N., & Tassin, B. (2015). Microplastic contamination in an urban area: a case study in Greater Paris. *Environ. Chem., 12*(5), 592-599.
9. Dyachenko, A., Mitchell, J., & Arsem, N. (2017). Extraction and identification of microplastic particles from secondary wastewater treatment plant (WWTP) effluent. *Anal. Methods, 9*(9), 1412-1418.
10. Eriksen, M., Mason, S., Wilson, S., Box, C., Zellers, A., Edwards, W., . . . Amato, S. (2013). Microplastic pollution in the surface waters of the Laurentian Great Lakes. *Marine Pollution Bulletin, 77*(1-2), 177-182.
11. Estahbanati, S., & Fahrenfeld, N. (2016). Influence of wastewater treatment plant discharges on microplastic concentrations in surface water. *Chemosphere, 162*, 277-284.
12. Faure, F., Demars, C., Wieser, O., Kunz, M., & De Alencastro, L. (2015). Plastic pollution in Swiss surface waters: Nature and concentrations, interaction with pollutants. *Environmental Chemistry, 12*(5), 582-591.
13. Fischer, E., Paglialonga, L., Czech, E., & Tamminga, M. (2016). Microplastic pollution in lakes and lake shoreline sediments - A case study on Lake Bolsena and Lake Chiusi (central Italy). *Environmental Pollution, 213*, 648-657.
14. Free, C., Jensen, O., Mason, S., Eriksen, M., Williamson, N., & Boldgiv, B. (2014). High-levels of microplastic pollution in a large, remote, mountain lake. *Marine Pollution Bulletin, 85*(1), 156-163.
15. Hendrickson, E., Minor, E., & Schreiner, K. (2018). Microplastic Abundance and Composition in Western Lake Superior As Determined via Microscopy, Pyr-GC/MS, and FTIR. *Environmental Science and Technology, 52*(4), 1787-1796.
16. Hoellein, T., McCormick, A., Hittie, J., London, M., Scott, J., & Kelly, J. (2017). Longitudinal patterns of microplastic concentration and bacterial assemblages in surface and benthic habitats of an urban river. *Freshwater Science, 36*(3), 491-507.
17. Kosuth, M., Mason, S., & Wattenberg, E. (2018). Anthropogenic contamination of tap water, beer, and sea salt. *PLoS ONE, 13*(4).
18. Lahens, L., Strady, E., Kieu-Le, T., Dris, R., Boukerma, K., Rinnert, E., . . . Tassin, B. (2018). Macroplastic and microplastic contamination assessment of a tropical river (Saigon River, Vietnam) transversed by a developing megacity. *Environmental Pollution, 236*, 661-671.
19. Lares, M., Ncibi, M., Sillanpää, M., & Sillanpää, M. (2018). Occurrence, identification and removal of microplastic particles and fibers in conventional activated sludge process and advanced MBR technology. *Water Research, 133*, 236-246.
20. Leslie, H., Brandsma, S., van Velzen, M., & Vethaak, A. (2017). Microplastics en route: Field measurements in the Dutch river delta and Amsterdam canals, wastewater treatment plants, North Sea sediments and biota. *Environment International, 101*, 133-142.
21. Magnusson, K., & Norén, F. (2014). Screening of microplastic particles in and down-stream a wastewater treatment plant. *IVL Swedish Environmental Research Institute, C 55*, 22.
22. Mani, T., Hauk, A., Walter, U., & Burkhardt-Holm, P. (2015). Microplastics profile along the Rhine River. *Scientific Reports, 5*.
23. Mason, S., Garneau, D., Sutton, R., Chu, Y., Ehmann, K., Barnes, J., . . . Rogers, D. (2016). Microplastic pollution is widely detected in US municipal wastewater treatment plant effluent. *Environmental Pollution, 218*, 1045-1054.
24. Mason, S., Kammin, L., Eriksen, M., Aleid, G., Wilson, S., Box, C., . . . Riley, A. (2016). Pelagic plastic pollution within the surface waters of Lake Michigan, USA. *Journal of Great Lakes Research, 42*(4), 753-759.
25. Mason, S., Welch, V., & Neratko, J. (2018). *Synthetic polymer contamination in bottled water.*
26. McCormick, A., Hoellein, T., London, M., Hittie, J., Scott, J., & Kelly, J. (2016). Microplastic in surface waters of urban rivers: Concentration, sources, and associated bacterial assemblages. *Ecosphere, 7*(11).
27. McCormick, A., Hoellein, T., Mason, S., Schluep, J., & Kelly, J. (2014). Microplastic is an abundant and distinct microbial habitat in an urban river. *Environmental Science and Technology, 48*(20), 11863-11871.
28. Michielssen, M., Michielssen, E., Ni, J., & Duhaime, M. (2016). Fate of microplastics and other small anthropogenic litter (SAL) in wastewater treatment plants depends on unit processes employed. *Environmental Science: Water Research and Technology, 2*(6), 1064-1073.
29. Miller, R., Watts, A., Winslow, B., Galloway, T., & Barrows, A. (2017). Mountains to the sea: River study of plastic and non-plastic microfiber pollution in the northeast USA. *Marine Pollution Bulletin, 124*(1), 245-251.
30. Mintenig, S., Int-Veen, I., Löder, M., Primpke, S., & Gerdts, G. (2017). Identification of microplastic in effluents of waste water treatment plants using focal plane array-based micro-Fourier-transform infrared imaging. *Water Research, 108*, 365-372.
31. Mintenig, S., Löder, M., Primpke, S., & Gerdts, G. (2019). Low numbers of microplastics detected in drinking water from ground water sources. *Science of the Total Environment*.
32. Murphy, F., Ewins, C., Carbonnier, F., & Quinn, B. (2016). Wastewater Treatment Works (WwTW) as a Source of Microplastics in the Aquatic Environment. *Environmental Science and Technology, 50*(11), 5800-5808.
33. Oßmann, B., Sarau, G., Holtmannspötter, H., Pischetsrieder, M., Christiansen, S., & Dicke, W. (2018). Small-sized microplastics and pigmented particles in bottled mineral water. *Water Research*.
34. Pivokonsky, M., Cermakova, L., Novotna, K., Peer, P., Cajthaml, T., & Janda, V. (2018). Occurrence of microplastics in raw and treated drinking water. *Science of the Total Environment*.
35. Rodrigues, M., Abrantes, N., Gonçalves, F., Nogueira, H., Marques, J., & Gonçalves, A. (2018). Spatial and temporal distribution of microplastics in water and sediments of a freshwater system (Antuã River, Portugal). *Science of the Total Environment, 633*, 1549-1559.
36. Schymanski, D., Goldbeck, C., Humpf, H., & Fürst, P. (2018). Analysis of microplastics in water by micro-Raman spectroscopy: Release of plastic particles from different packaging into mineral water. *Water Research, 129*, 154-162.
37. Sighicelli, M., Pietrelli, L., Lecce, F., Iannilli, V., Falconieri, M., Coscia, L., . . . Zampetti, G. (2018). Microplastic pollution in the surface waters of Italian Subalpine Lakes. *Environmental Pollution, 236*, 645-651.
38. Simon, M., Alst, N., Vollertsen, J., Simon, M., van Alst, N., & Vollertsen, J. (2018). Quantification of microplastic mass and removal rates at wastewater treatment plants applying Focal Plane Array (FPA)-based Fourier Transform Infrared (FT-IR) imaging. *Water Research, 142*, 1-9.
39. Su, L., Xue, Y., Li, L., Yang, D., Kolandhasamy, P., Li, D., & Shi, H. (2016). Microplastics in Taihu Lake, China. *Environmental Pollution, 216*, 711-719.
40. Talvitie, J., Heinonen, M., Pääkkönen, J., Vahtera, E., Mikola, A., Setälä, O., & Vahala, R. (2015). Do wastewater treatment plants act as a potential point source of microplastics? Preliminary study in the coastal Gulf of Finland, Baltic Sea. *Water Science and Technology, 72*(9), 1495-1504.
41. Talvitie, J., Mikola, A., Koistinen, A., & Setälä, O. (2017). Solutions to microplastic pollution – Removal of microplastics from wastewater effluent with advanced wastewater treatment technologies. *Water Research, 123*, 401-407.
42. Talvitie, J., Mikola, A., Setï¿½lï¿½, O., Heinonen, M., & Koistinen, A. (2017). How well is microlitter purified from wastewater? – A detailed study on the stepwise removal of microlitter in a tertiary level wastewater treatment plant. *Water Research, 109*, 164-172.
43. Vermaire, J., Pomeroy, C., Herczegh, S., & Haggart, O. (2017). Microplastic abundance and distribution in the open water and sediment of the Ottawa River, Canada, and its tributaries. *Facets, 2*(1), 301-314.
44. Vollertsen, J., & Hansen, A. (2017). Microplastic in Danish wastewater Sources, occurrences and fate.
45. Wang, W., Ndungu, A., Li, Z., & Wang, J. (2017). Microplastics pollution in inland freshwaters of China: A case study in urban surface waters of Wuhan, China. *Science of the Total Environment, 575*, 1369-1374.
46. Wang, W., Yuan, W., Chen, Y., & Wang, J. (2018). Microplastics in surface waters of Dongting Lake and Hong Lake, China. *Science of the Total Environment, 633*, 539-545.
47. Xiong, X., Zhang, K., Chen, X., Shi, H., Luo, Z., & Wu, C. (2018). Sources and distribution of microplastics in China's largest inland lake – Qinghai Lake. *Environmental Pollution, 235*, 899-906.
48. Zhang, K., Gong, W., Lv, J., Xiong, X., & Wu, C. (2015). Accumulation of floating microplastics behind the Three Gorges Dam. *Environmental Pollution, 204*, 117-123.
49. Zhang, K., Xiong, X., Hu, H., Wu, C., Bi, Y., Wu, Y., . . . Liu, J. (2017). Occurrence and Characteristics of Microplastic Pollution in Xiangxi Bay of Three Gorges Reservoir, China. *Environmental Science and Technology, 51*(7), 3794-3801.
50. Ziajahromi, S., Neale, P., Rintoul, L., & Leusch, F. (2017). Wastewater treatment plants as a pathway for microplastics: Development of a new approach to sample wastewater-based microplastics. *Water Research, 112*, 93-99.


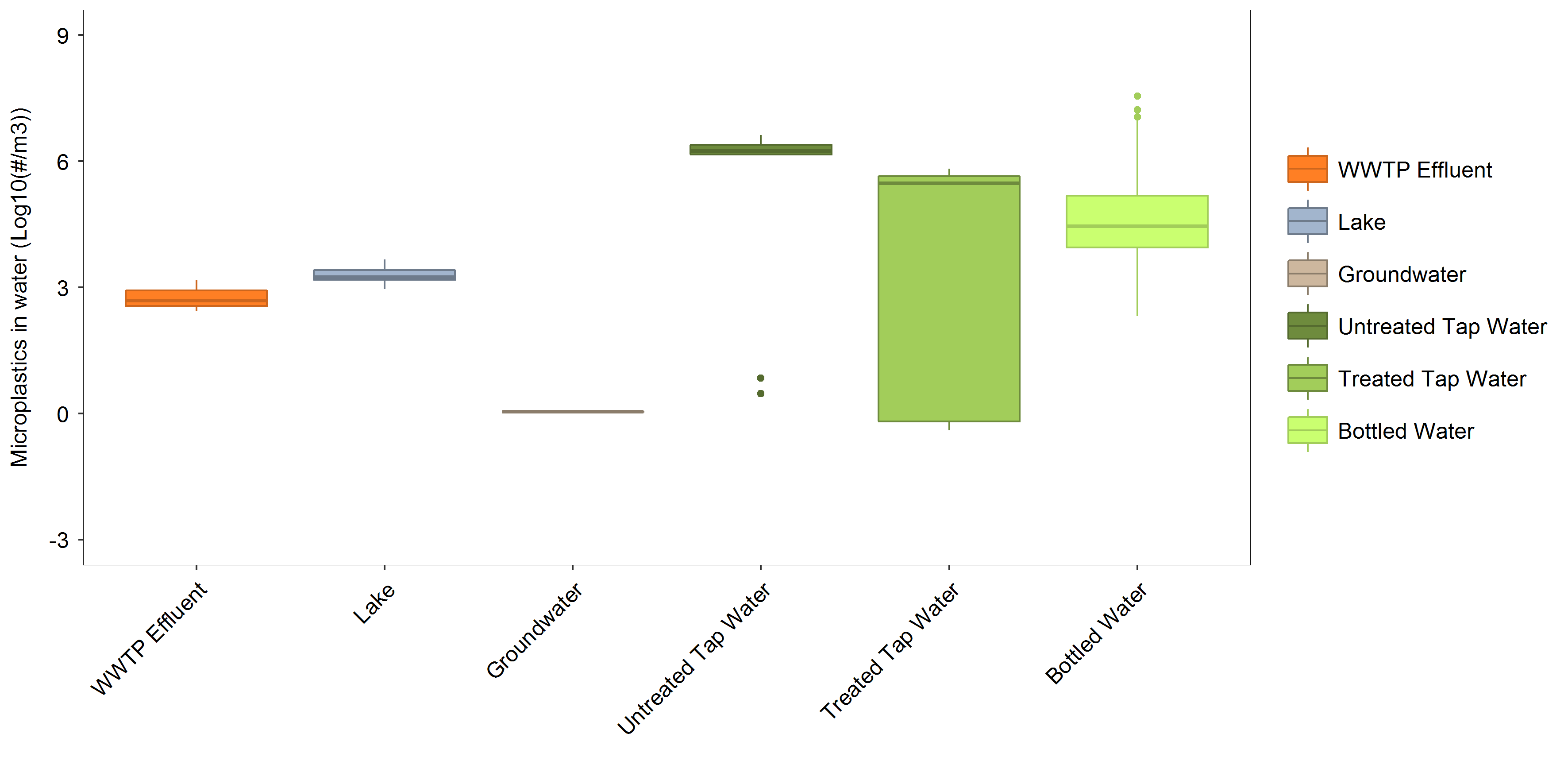


**Figure S1**. Box and whisker plot showing median and variation in microplastic number concentrations in individual samples taken from different water types. Data relate to individual samples unless only means were reported, in which case the mean value was taken into account n times, with n being the number of samples which the mean was based on. Only studies reporting number concentration with highest reliability scores were included (Wang et al. 2018; Mason et al., 2018; Ziajahromi et al. 2017). Additionally, data from four studies that only lacked positive controls were included (Ossman et al., 2018; Schymanski et al., 2018; Mintenig et al. 2019; Pivokonsky et al. 2018).
